# Stability of gene expression in human T cells in different gravity environments is clustered in chromosomal region 11p15.4

**DOI:** 10.1038/s41526-017-0028-6

**Published:** 2017-08-31

**Authors:** Cora S. Thiel, Andreas Huge, Swantje Hauschild, Svantje Tauber, Beatrice A. Lauber, Jennifer Polzer, Katrin Paulsen, Hartwin Lier, Frank Engelmann, Burkhard Schmitz, Andreas Schütte, Liliana E. Layer, Oliver Ullrich

**Affiliations:** 1 Institute of Anatomy, Faculty of Medicine, University of Zurich, Winterthurerstrasse 190, CH-8057 Zurich, Switzerland; 2 Department of Machine Design, Engineering Design and Product Development, Institute of Mechanical Engineering, Otto-von-Guericke-University Magdeburg, Universitätsplatz 2, D-39106 Magdeburg, Germany; 3 Core Facility Genomic, Medical Faculty of Muenster, University of Muenster, Albert-Schweitzer-Campus 1, D3, Domagstrasse 3, D-48149 Muenster, Germany; 4 KEK GmbH, Kemberger Str. 5, D-06905 Bad Schmiedeberg, Germany; 5 Ernst-Abbe-Hochschule Jena, Carl-Zeiss-Promenade 2, D-07745 Jena, Germany; 6 Airbus Defence and Space, Airbus DS GmbH, D-28199 Bremen, Germany; 7 Zurich Center for Integrative Human Physiology (ZIHP), University of Zurich, Winterthurerstrasse 190, CH-8057 Zurich, Switzerland; 8 Institute of Space Life Sciences, School of Life Sciences, Beijing Institute of Technology, Beijing, 100081 China

## Abstract

In the last decades, a plethora of in vitro studies with living human cells contributed a vast amount of knowledge about cellular and molecular effects of microgravity. Previous studies focused mostly on the identification of gravity-responsive genes, whereas a multi-platform analysis at an integrative level, which specifically evaluates the extent and robustness of transcriptional response to an altered gravity environment was not performed so far. Therefore, we investigated the stability of gene expression response in non-activated human Jurkat T lymphocytic cells in different gravity environments through the combination of parabolic flights with a suborbital ballistic rocket and 2D clinostat and centrifuge experiments, using strict controls for excluding all possible other factors of influence. We revealed an overall high stability of gene expression in microgravity and identified olfactory gene expression in the chromosomal region 11p15.4 as particularly robust to altered gravity. We identified that classical reference genes *ABCA5*, *GAPDH*, *HPRT1*, *PLA2G4A*, and *RPL13A* were stably expressed in all tested gravity conditions and platforms, while *ABCA5* and *GAPDH* were also known to be stably expressed in U937 cells in all gravity conditions. In summary, 10–20% of all transcripts remained totally unchanged in any gravitational environment tested (between 10^−4^ and 9 g), 20–40% remained unchanged in microgravity (between 10^−4^ and 10^−2^ g) and 97–99% were not significantly altered in microgravity if strict exclusion criteria were applied. Therefore, we suppose a high stability of gene expression in microgravity. Comparison with other stressors suggests that microgravity alters gene expression homeostasis not stronger than other environmental factors.

## Gene expression: potentially low-risk for long-term space missions

Approximately 99% of genes in human lymphocytic cells have the same transcription activity on Earth as they do in microgravity environments. An international team led by Oliver Ullrich and Cora Thiel at the University of Zurich in Switzerland used a combination of parabolic aircraft and suborbital rocket flights and ground-based experiments to show how gene expression is stable through a range of gravity conditions. Screening of microarrays identified several reference genes that may serve as standardized controls for future immune cell trials. Their experiments also revealed that the chromosomal region associated with olfactory and taste receptor genes is particularly robust to altered gravity. Because altered transcripts were associated with fast cellular adaptation, the researchers predict that the risk of unusual gene behavior will be quite low during extended space deployments.

## Introduction

Life is adapted to the gravitational force on Earth and physical, chemical, and biological processes in organisms and cells have evolved under the influence of this natural constant. Although many limiting factors for human health and performance in microgravity have been identified,^1^ long-term space flights for up to several months are meanwhile established since decades during International Space Station (ISS) missions. Therefore, it can be expected that human cells must have sufficient and sustainable mechanisms allowing life under non-terrestrial microgravity conditions.

The immune system belongs to the affected systems during spaceflight^1^ and sensitivity of cells of the human immune system to reduced gravity has been confirmed by numerous studies in real and simulated microgravity.^2–5^ The first space experiment which investigated the effects of altered gravity in isolated cells of the human immune system found not only a <3% induction of activation upon stimulation with Concanavalin A in lymphocytes exposed to microgravity, but also an almost doubled proliferation rate when exposing lymphocytes to 10 g instead of 1 g.^6^ These findings made very clear that cells are in principle sensitive to gravity, not only in the case of reduced gravity, but also increased gravity. Unfortunately, the mechanisms underlying gravity signal transduction still remain unknown, although several studies confirmed differential gene expression patterns of lymphocytes subjected to altered gravity.

In peripheral blood lymphocytes, the expression of *IL2* and *IL2 receptor alpha* was shown to be significantly reduced in simulated microgravity,^7^ as well as *PKC* isoforms *delta* and *epsilon*.^8^ In the former study, *beta-actin* was supposed to be stably expressed and used for normalization of real-time (RT) PCR experiments, while in the latter study, *GAPDH* expression was supposed to be not compromised by altered gravity and was used for normalization. By microarray analysis, a downregulation of the PKA pathway and 91 genes with reduced expression in simulated microgravity were found, many of them being early genes of activation.^9^ Another study identified 89 genes demonstrating altered expression in modeled microgravity of which 79 were down and 10 were upregulated comprising genes related to immune response, signal transduction, apoptosis, tissue growth regulation, DNA repair, and others.^10^ Furthermore, microarray analysis revealed a total of 122 genes being affected by gravity alteration, e.g., downregulation of T-cell activation genes and upregulation of *IL4 receptor*.^11^ In CD3/CD28-stimulated primary human T cells and in Jurkat T cells rapid alterations of the cell cycle control protein *p21* were observed in real microgravity,^12^ while another study observed inhibition of the Rel/NF-κB pathway by microgravity and of 47 genes, including immediate early genes in T-cell activation showing that microgravity was the causative factor for impaired T-cell activation during spaceflight.^13^ In the latter, *cyclophilin* was regarded as stable under altered gravity conditions and was used for normalization in quantitative RT PCR (qRT-PCR).

Using leukemic Jurkat T cells and complimentary DNA microarray analysis, 11 cytoskeleton-related genes, including *calponin*, *dynactin*, *tropomodulin*, *keratin 8*, two *myosins*, an ankyrin EST, an actin-like protein, the cytoskeletal linker plectin, and a centriole-associated protein (*C-NAP1*) were found upregulated in spaceflight microgravity, while *gelsolin precursor* was downregulated.^14^ *Beta-actin* was assumed to be stably expressed in microgravity compared to normogravity and used for normalization in qRT-PCR.^14^ In another lymphoblast leukemic cell line (MOLT-4), *CDK1* and the *MYC* genes were found downregulated in microgravity, while the receptor tyrosine kinase c-kit/CD117 and the immediate early gene *JUNB* were upregulated.^15^ Furthermore, MOLT-4 cultures differentially expressed a total of 349 up and 444 downregulated genes under microgravity.^15^ Again, *beta-actin* and *U6* were regarded as stably expressed under altered gravity conditions and were used for normalization in qRT-PCR experiments.

Studying spaceflight effects on T lymphocyte distribution, function, and gene expression in murine cells from spleen, a decrease of *IL2* and an increase of *IL10*, *IFN-gamma*, and *MIP-1 alpha* after activation with anti-CD3 antibody were observed.^16^ Microarray analysis of murine thymus tissue after spaceflight further revealed differential expression in genes regulating stress and glucocorticoid receptors as *Rbm3* (upregulated), and *Hsph110*, *Hsp90aa1, Cxcl10, Stip1*, and *Fkbp4* (downregulated).^17^ qRT-PCR demonstrated additional gene expression alteration in other T-cell related genes, including *Ctla4*, *Ifna 2a* (upregulated), and *Cd44* (downregulated).^17^ Gene expression changes in mouse thymus and spleen were also investigated in a further study during space shuttle STS-135 mission in which normal cell genes *Il10*, *Il18bp*, *Il18r1*, and *Spp1* were upregulated, while *Ccl7*, *Il6* were downregulated, and cancer-related genes *Casp8*, *Fgfr2*, *Figf*, *Hgf*, *IGF1*, *Itga4*, *Ncam1*, *Pdgfa*, *Pik3r1*, *Serpinb2*, and *Sykb* were upregulated as well, whereas *Cdc25a*, *E2F1*, *Mmp9*, and *Myc* showed decreased expression in thymus.^18^ At the same time, spleen cells showed upregulation of cancer-related gene *Cdkn2a* during spaceflight, while *Birc5*, *Casp8*, *Ctnnb1*, *Map2k1*, *Mdm2*, *Nfkb1*, and *Pdgfa* displayed less expression.^18^ Another study demonstrating significantly impaired mouse immune function in spaceflight and simulated microgravity revealed a significant reduction of key gene expression as *IL2, IL2 receptor alpha, IFN-gamma*, and *Tagap* during early T-cell activation of spleenocytes.^19^ Apart from that, two new early T-cell activation genes were identified, *Iigp1* and *Slamf1*, showing reduced expression under microgravity as well. *Cyclophilin* expression was used for normalization in qRT-PCR in that study and was therefore regarded as stably expressed.

Recently, also *microRNA-21* was found differentially expressed during T-cell activation under spaceflight conditions exhibiting upregulation in normal gravity, while being suppressed under microgravity together with 85 other genes.^20^ In that study, *cyclophilin A* was regarded stable between normal gravity and microgravity conditions, and therefore used for normalization in qRT-PCR experiments.^20^ Simulated microgravity revealed altered expression of microRNA in human lymphoblastoid TK6 cells as well, including *miR-150, miR-34a, miR-423-5p, miR-22, miR-141, miR-618*, and *miR-222*, while *beta-actin* and *GAPDH* were assumed to be unchanged and therefore used for normalization.^21^ Moreover, the altered miRNA expression was confirmed to correlate with gene expression of several transcription factors including *EGR2, ETS1*, and *REL*.^21^

In summary, a plethora of coordinated in vitro studies with living human cells in microgravity, experiments on board of parabolic flights, suborbital or orbital flights, and with ground-based facilities (GBFs) for simulated microgravity, contributed a vast amount of knowledge and brought us very close to the potential primary cellular and molecular mechanisms behind the effects of altered gravity. Previous studies focused mostly on the identification of gravity-responsive genes, whereas a multi-platform analysis at an integrative level, which specifically evaluates the extent and robustness of transcriptional response to an altered gravity environment was not performed so far. Therefore, to understand the extent gene expression response in an altered gravity environment at an integrative level, we investigated systematically non-changing genes, whose homeostasis is not affected by the gravitational force. Interestingly, one study reported that of the more than 20,000 genes interrogated, ~98% were similarly expressed in flight and ground controls, while <2% were expressed more in space and only a few were downregulated.^14^ These numbers indicate that in spite of the numerous reports of differential gene expression under altered gravity, the actual dysregulation or regulation response of the genome in altered gravity may not be that extensive after all. In our study, we used microarray analysis to investigate differential gene expression in Jurkat cells, a lymphocytic human cell line, exposed to short-term (20 s), mid-term (5 min), and simulated (5 min) microgravity during parabolic flights, suborbital ballistic rocket flight, and during 2D clinostat experiments. Additionally, cells were exposed to hypergravity before the microgravity phases in the parabolic and the sounding rocket flights as well as on a 9 g centrifuge. Our aim was to identify in particular the stable gene transcripts and to estimate the magnitude of gene expression alteration in order to better understand the adaptation processes and pathways in different gravity environments.

Within the scope of this investigation and by comparing the results to former studies in monocytic cells,^22^ we also aimed at identifying reliable and comprehensive reference genes for differential gene expression analysis performed with immune cells on the major Earth-based platforms providing real and simulated microgravity. Reference genes (“housekeeping genes”) are essential to verify and standardize gene expression analysis as internal controls, also for the amount and quality of starting material and the reaction efficiency. Although numerous publications described differential gene expression analysis in T lymphocytes under simulated and real microgravity conditions in various cells types and tissues,^7–21^ a systematic analysis of suitable reference genes has not been demonstrated so far.

Therefore, we also re-evaluated 20 reference genes identified earlier as widely applied throughout the literature^22^ using microarray data for these particular genes in human Jurkat T lymphocytes^23^ and assessed their stability.^23, 24^ Finally, our study aims to estimate to which extent changes of the gravity environment influence gene expression homeostasis compared to other environmental stress factors and to provide therefore new data for an appropriate risk assessment of long-term space missions.

## Results

Aim of this study was to investigate gene expression stability in a human cell line in different gravity environments through the combination of parabolic flights with a suborbital ballistic rocket experiment and 2D clinostat and centrifuge experiments, and through the use of strict controls for excluding all possible other factors of influence. Our approach allowed the identification and validation of gravity-independent gene expression through two fully independent large-scale flight campaigns and one ground-based experiment campaign, in which sets of independent experiments were conducted. Therefore, transcriptional stability identified after these experiment campaigns are characterized by a high level of evidence, not only due to several independent experiments, but also due to independent research platforms.

### Different independent research platforms allow cross-validation of experiment results

**Fig. 1:**
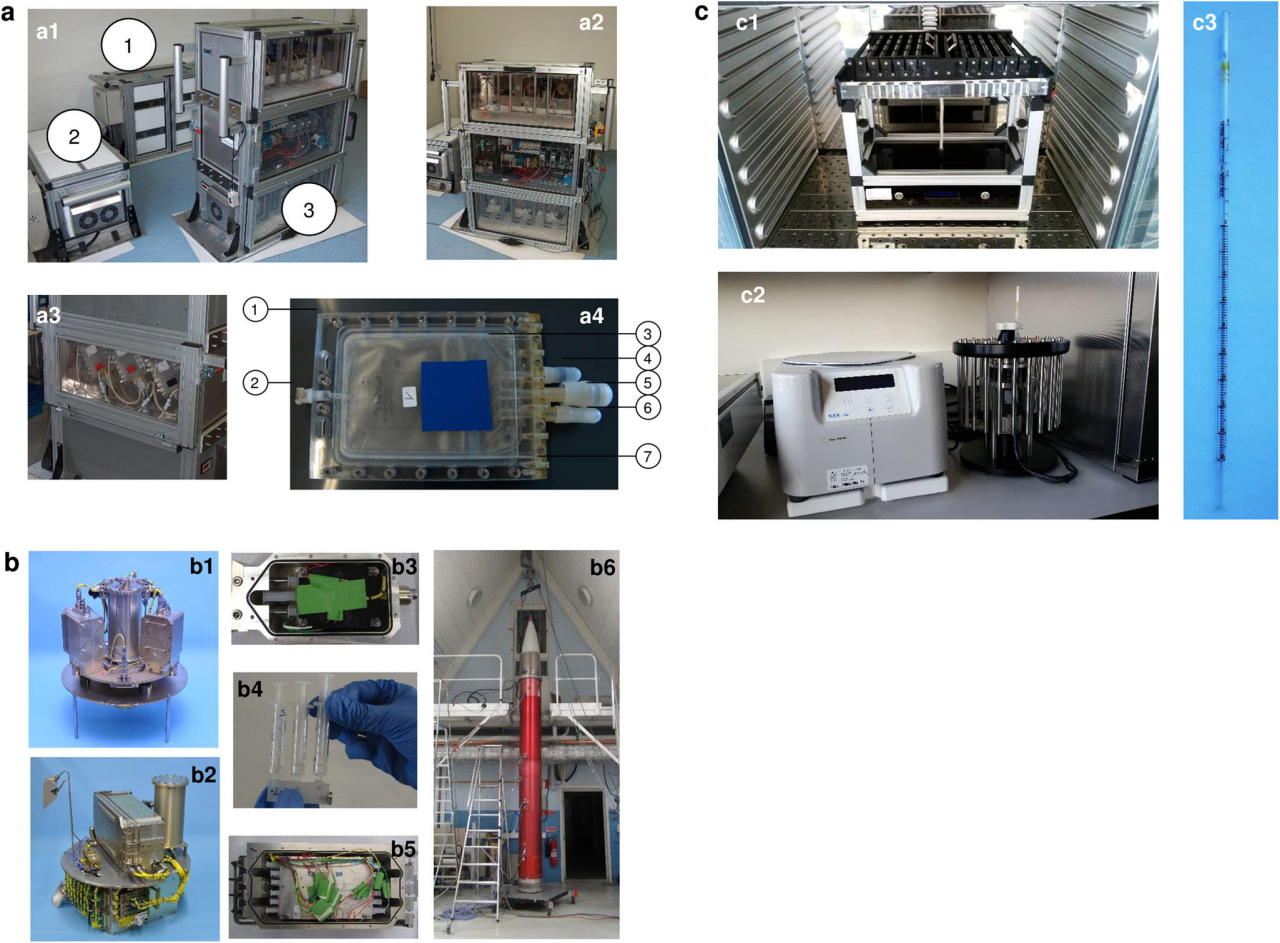
Experiment hardware of the parabolic flight (23rd DLR PFC), suborbital rocket (TEXUS-51), and ground-based experiments.** a** In-flight experiment system for parabolic flights on board the Airbus A300 ZERO-G. a1 Experiment hardware structure that consists of an incubator rack to store the cell containers at 37 °C before the experiment (1), an experiment rack, in which all technical aggregates are accommodated for the execution of the experiment and where the living cells are processed during altered gravity (2), and a cooling rack to store all cell containers at 4 °C after the injection of the lysis solution until landing (3). a2 Structure of the working rack, *rear side*. In the *upper* *third* (4 °C) are three separate hose pumps, which pump the lysis solution into the cell containers, controlled by the unit inside the *middle third*, which also carries all electrical connections and fuse elements. All liquids are sucked under exclusion of air. In the *lower part* (36.5 °C) are three separate hose pumps, which pump the medium into the cell containers. a3 Structure of the working rack, *front side*, waterproof working space with cell containers. a4 Double-walled, liquid-proof cell container. A maximum of 54 containers can be accommodated during one flight. 1 = plastic container, 2 = air valve, 3 = internal sterile cell culture bag (Nutrimix, 0.25 l), 4 = connector 1 (medium), 5 = connector 2 (lysis buffer), 6 = connector 3 (port for filling of cells, performed pre-flight), 7 = plastic flange. **b** In-flight experiment system for the suborbital ballistic rocket flight of the TEXUS-51 payload. TEXUS consists of a VSB-30 engine (not shown) and of the payload structure (b6). Sets of three sterile syringes were filled with cell suspension, medium, and lysis buffer connected by a T-piece with small plugs at the outlet ports to prevent premature contact of the fluids (b4). The syringe systems are accommodated in tempered and vacuum-resistant containers (b3, b5) at the static (b2) or centrifuge (b1) position. **c** Ground-based facilities. c1 Fast rotating 2D clinostat for pipettes. Jurkat T lymphocytes in cell culture medium were filled into sterile plastic pipettes with a diameter of 3.5 mm (c3), sealed and installed on the clinostat located in a 37 °C incubator. Samples were lysed after 300 s rotation time at 60 rpm. c2 Centrifuge for pipettes. Jurkat T cells in suspension were filled in sterile plastic pipettes with a diameter of 3.5 mm (c3), pipettes were sealed with a sterile rubber plug and were placed into the pipette holders of the centrifuge. Samples were lysed after the centrifuge run at maximum speed at 9 g for 300 s at 37 °C

**Fig. 2:**
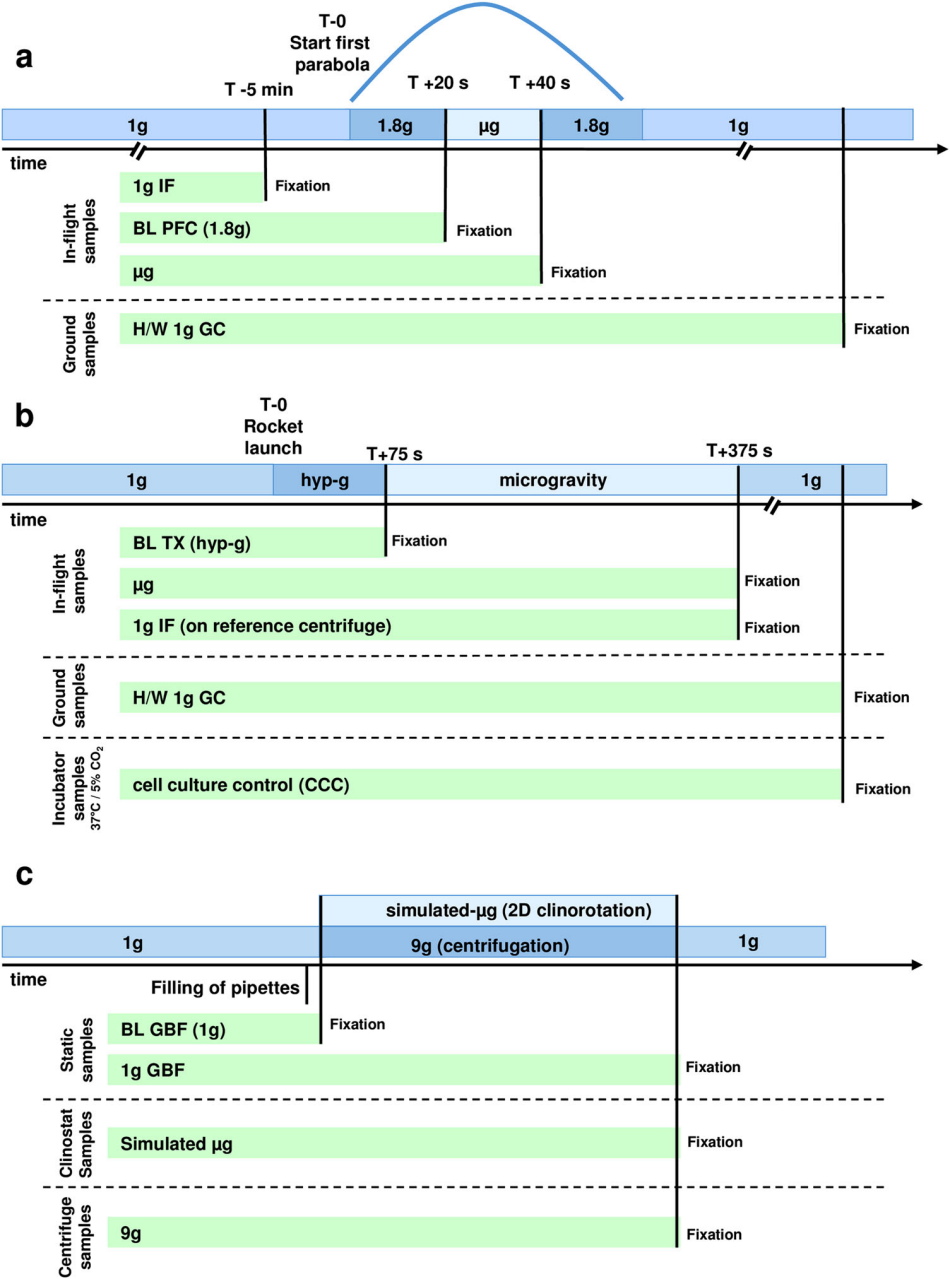
Sample lysis scheme. **a** Jurkat T cells were analyzed during the 23rd DLR parabolic flight campaign. In total, four sample groups were lysed at defined g conditions and time points: (1) 1 g in-flight (*1 g IF*) 5 min before the first parabola, (2) 1.8 g samples at the end of the first parabola after 20 s of the 1.8 g hypergravity phase; these samples serve also as baseline (BL-PFC) directly before the microgravity phase, (3) at the end of the first parabola after 20 s microgravity (*μg*) phase, and (4) 1 g hardware ground control (*H/W* 1 g *GC*), directly after the flight inside the aircraft. **b** Jurkat T lymphocytes were investigated during the TEXUS-51 sounding rocket campaign. Overall, five sample groups were lysed at set time points and g conditions: (1) the baseline (BL-TX) group monitored the first 75 s of the flight after liftoff including hypergravity and vibrations, (2) microgravity samples were lysed 375 s post-launch, resulting in 300 s of microgravity time, (3) 1 g IF controls inside a reference centrifuge experienced 300 s 1 g simultaneously to the microgravity samples, (4) 1 g H/W GC lysed ~15 min after launch together with (5) the cell culture controls as reference for effects originating from the exposure of the cells to the hardware. **c** Jurkat T cells were examined in ground-based facilities (*GBFs*). Four groups were investigated in total: (1) the baseline (BL-GBF) group that monitored the 1 g situation, especially the influence of shear forces during the aspiration of the cells in the pipette and the following efflux, (2) H/W 1 g GC for which cells were filled in the pipettes and incubated for 300 s at the static base of the clinostat at 37 °C, (3) simulated microgravity group (*sim-μg*), cells were clinorotated for 300 s at 37 °C, and (4) the hypergravity group, Jurkat lymphocytes were centrifuged at 9 g for 300 s before lysis

In this study, we investigated and compared the whole-genome expression of genes in T lymphocytes on different microgravity platforms. We focused on those genes, gene families, or functional gene clusters in T cells that remained stable under altered gravity conditions. The following platforms were used: (1) parabolic flights for short-term microgravity experiments (20 s), (2) suborbital ballistic rocket experiment for mid-term microgravity investigations (5 min), and (3) 2D clinostat and centrifuge experiments to complement the experiments (Fig. 1). Jurkat T lymphocytes were cultivated for all experiments according to a standardized protocol and loaded into the corresponding hardware shortly before the experiment was performed. In the case of parabolic flights, experiments were executed only during the first parabola on three different flight days in order to ensure that cells were not subjected to any microgravity phase before the experiment. Following experimental groups were investigated during the 23rd DLR (German Aerospace Center) parabolic flight campaign (PFC): hardware 1 g ground control, 1 g in-flight control, 1.8 g hypergravity, (representing the baseline status immediately before the first microgravity phase), and the microgravity (μg) phase (Table 1; Fig. 2). The cells were exposed during the flight to 1.8 and μg for 20 s and lysed at the end of either phase. During the suborbital ballistic rocket flight on the TEXUS-51 mission, T lymphocytes were subjected to vibrations and up to 12.6 g hypergravity for max. 75 s after liftoff. Directly at the end of the hypergravity phase, dedicated samples were lysed, representing the baseline status immediately before the microgravity phase. A further sample set was lysed 300 s after onset of microgravity (=375 s after liftoff). Additional samples on a 1 g on board centrifuge represented the 1 g in-flight controls and were also lysed 300 s after onset of microgravity, so that a direct comparison of the microgravity flight samples with the 1 g in-flight controls allowed the immediate identification of the effects of altered gravity. Furthermore, a 1 g ground control group was analyzed, where the T cells were located in the hardware identical to the flight hardware and lysed simultaneously with the microgravity samples. For the identification of hardware-associated effects a cell culture control was also included in the experiment (Table 1; Fig. 2). A third set of experiments was performed for 300 s in a 2D pipette clinostat with calculated gravitational force of 4 × 10^−3^ g and in a pipette centrifuge with a gravitational force of 9 g. 1 g control experiments for both experiment platforms were placed at the base of the equipment for the duration of the experiment in order to control for non-gravity-associated hardware effects. Further baseline samples, where the cells were aspirated in the pipette and immediately dispensed and lysed, were used to control the status immediately before the simulated microgravity phase (Table 1; Fig. 2).

**Table 1:** Overview of microgravity platforms and ground-based facilities applied in different experimental research campaigns

		23rd DLR PFC	TEXUS-51	GBFs
Gravity condition	CCC 1 g ground	NA	+	NA
	H/W 1 g GC	+	+	NA
	1 g IF	+	+ (5 min, on centrifuge)	NA
	1 g GBF	NA	NA	1 g GBF (300 s)
	BL (directly before μg phase)	BL-PFC 1.8 g (20 s)	BL-TX (hyp-g) 1 g—max. 12.6 g (75 s)	BL-GBF 1 g (~10 s)
	Microgravity (μg)	μg (20 s)	μg (300 s)	sim μg (300 s clinorotation)
	Hypergravity (centrifuge)	NA	NA	9 g (300 s centrifugation)

Gravity conditions and times are given for parabolic flight, sounding rocket, and ground-based experiments. *BL* baseline, *CCC* cell culture control, *GBF* ground-based facility, *GC* ground control, *H/W* hardware, *IF* in-flight, *NA* not applicable, *PFC* parabolic flight campaign, *TX* TEXUS mission

### Sample processing and RNA quality

**Fig. 3:**
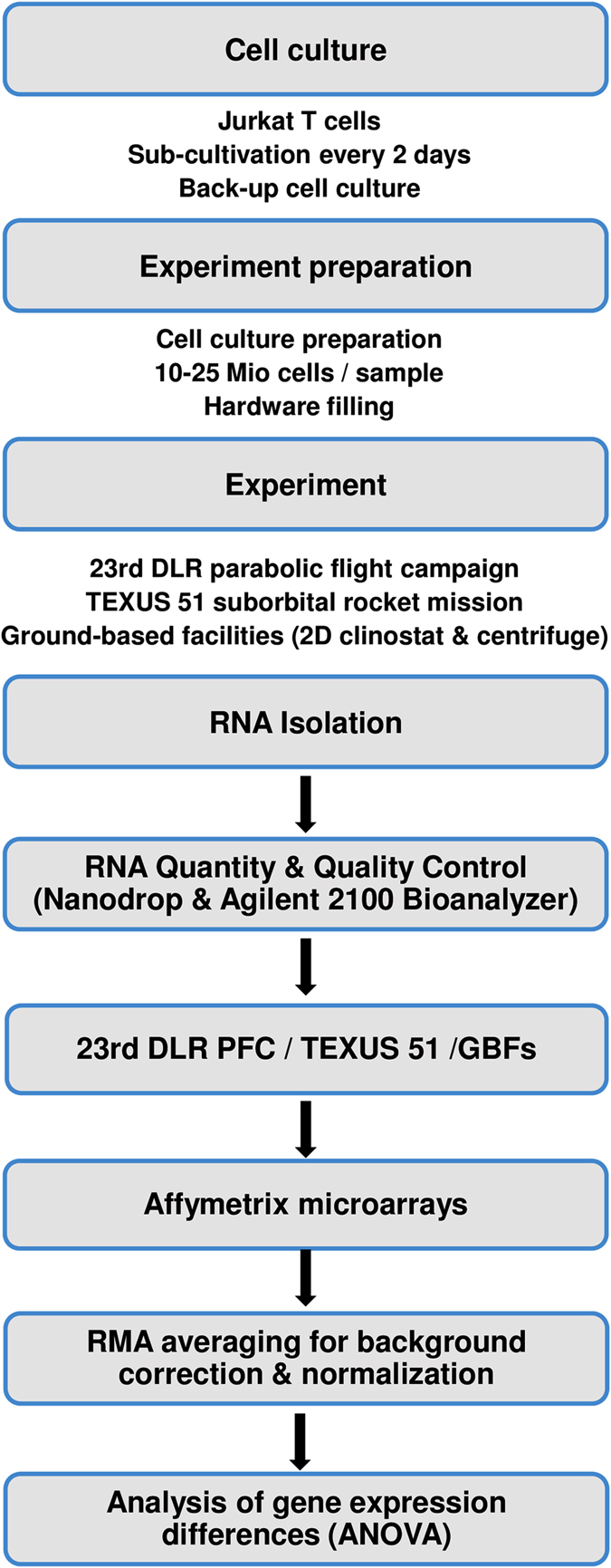
Experiment flow chart. Flow charts for experimental procedures for different platforms and cell types. *GBFs* ground-based facilities

For each experimental platform, RNA was isolated as fast as possible after completion of the experiment and the quantity and quality were determined. Subsequently, the RNA integrity number was determined and only RNAs with an RNA integrity number value higher than 8.2 and a concentration of more than 100 ng/μl were included in the microarray analysis. All RNA samples from Jurkat T cells originating from all three platforms were hybridized with the same Affymetrix GeneChip (Human Transcriptome Array 2.0) and analyzed with the Affymetrix expression and transcriptome analysis console. A standard data processing for background correction and normalization procedure was performed using the robust multi-array averaging method. Differential and stable gene expression analysis was identified on the basis of analysis of variance statistical testing. A flowchart of the experiment is shown in Fig. 3.

### Identification of reference genes in Jurkat T lymphocytes with stable gene expression in different gravity environments

**Fig. 4:**
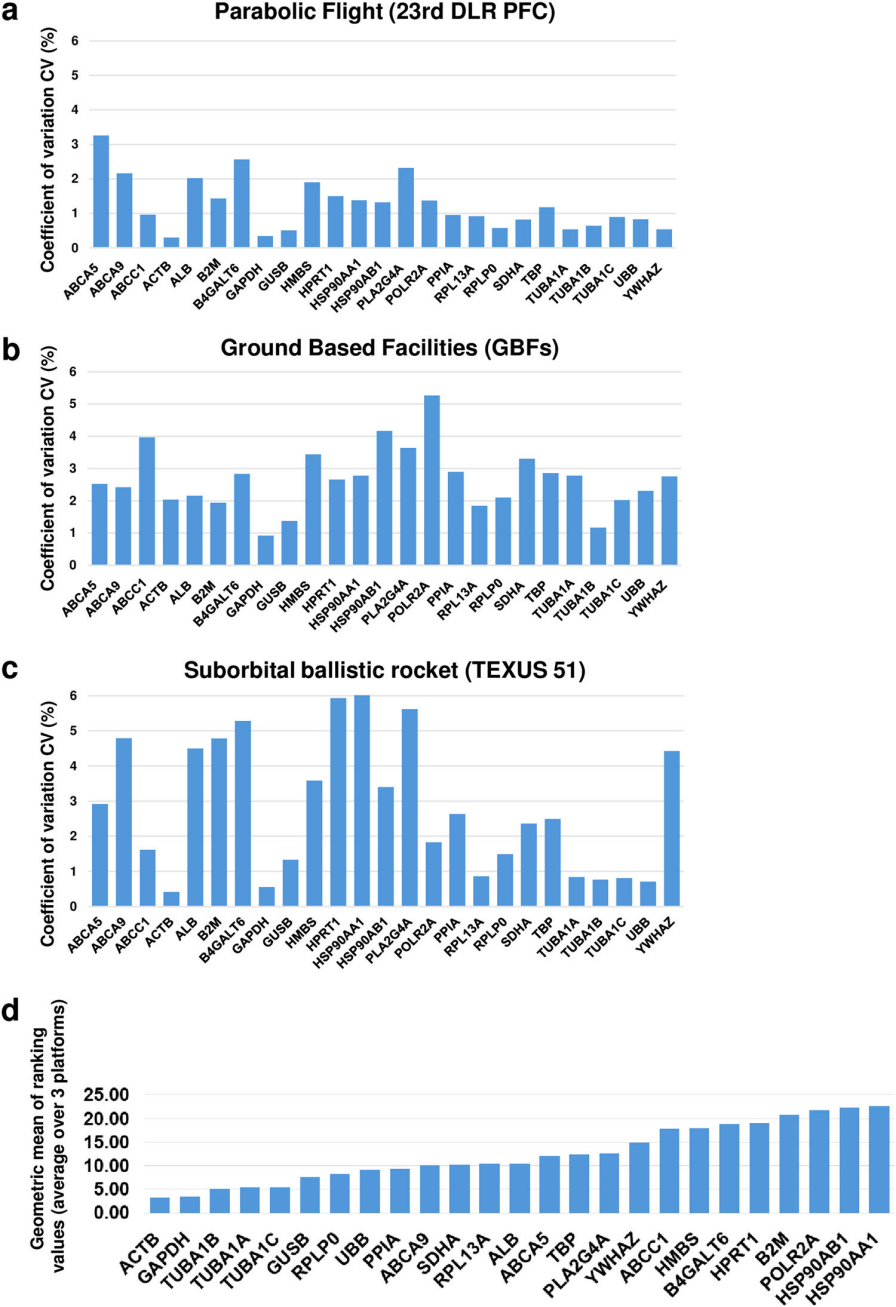
Coefficient of variation value and RefFinder analysis. The coefficients of variation values were calculated for all reference genes listed in Tables 2 and 3. All investigated genes show low coefficient of variation values and are therefore theoretically suited to serve as reference genes in expression experiments. Additionally, a RefFinder analysis was performed for the data of all genes originating from all three platforms and a ranking was calculated for the best reference genes. **a** parabolic flight (23rd DLR PFC), **b** ground-based facilities, **c** suborbital ballistic rocket (TEXUS-51), **d** geometric mean of ranking values (average over three platforms)

**Fig. 5:**
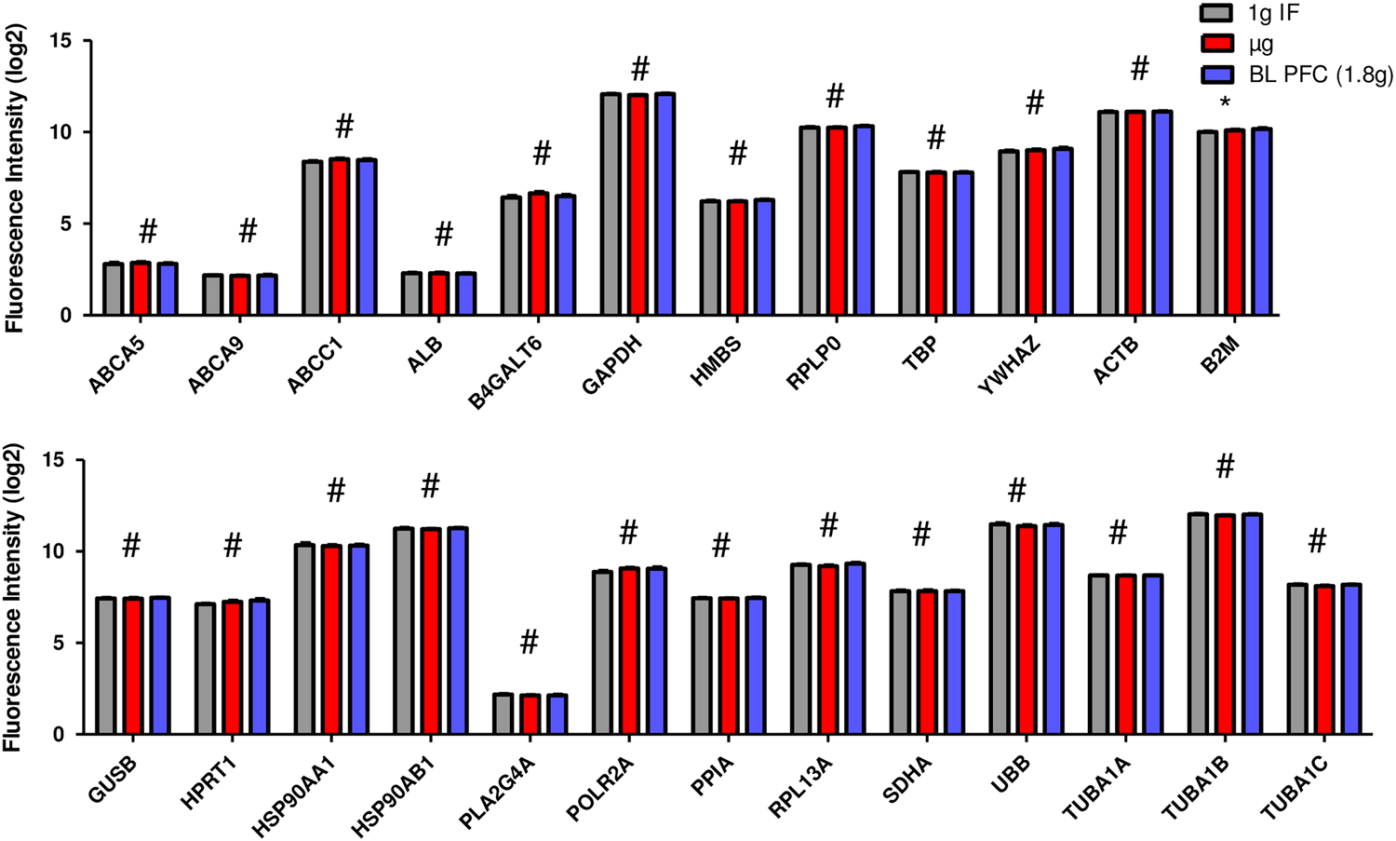
Microarray gene expression analysis of reference genes in samples exposed to different gravity conditions during parabolic flights. *Bars* represent mean log2 values of fluorescence intensities with SEM, *n* = 6. *1 g IF* 1 g in-flight control, *µg* microgravity samples, *BL-PFC* baseline parabolic flight campaign. * Significant differences in at least one group comparison, # no significant differences within the three groups according to one-way ANOVA with Tukey’s post-test

**Fig. 6:**
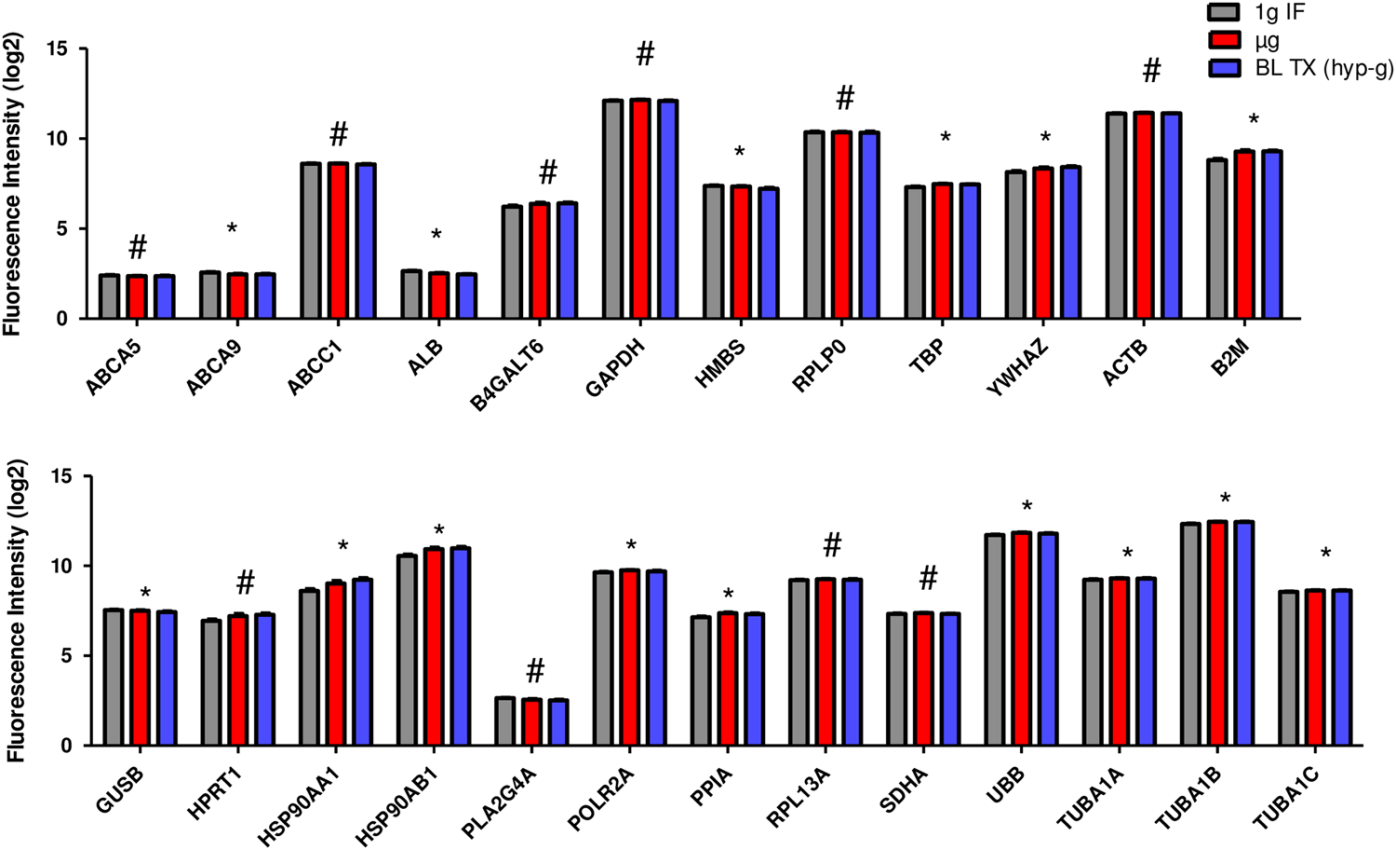
Microarray gene expression analysis of reference genes in samples exposed to different gravity conditions during the TEXUS-51 sounding rocket mission. *Bars* represent mean log2 values of fluorescence intensities with SEM, *n* = a minimum of seven samples *1 g IF* 1 g in-flight control, *µg* microgravity samples, *BL-TX* baseline TEXUS. * Significant differences in at least one group comparison, # no significant differences within the three groups according to one-way ANOVA with Tukey’s post-test

**Fig. 7:**
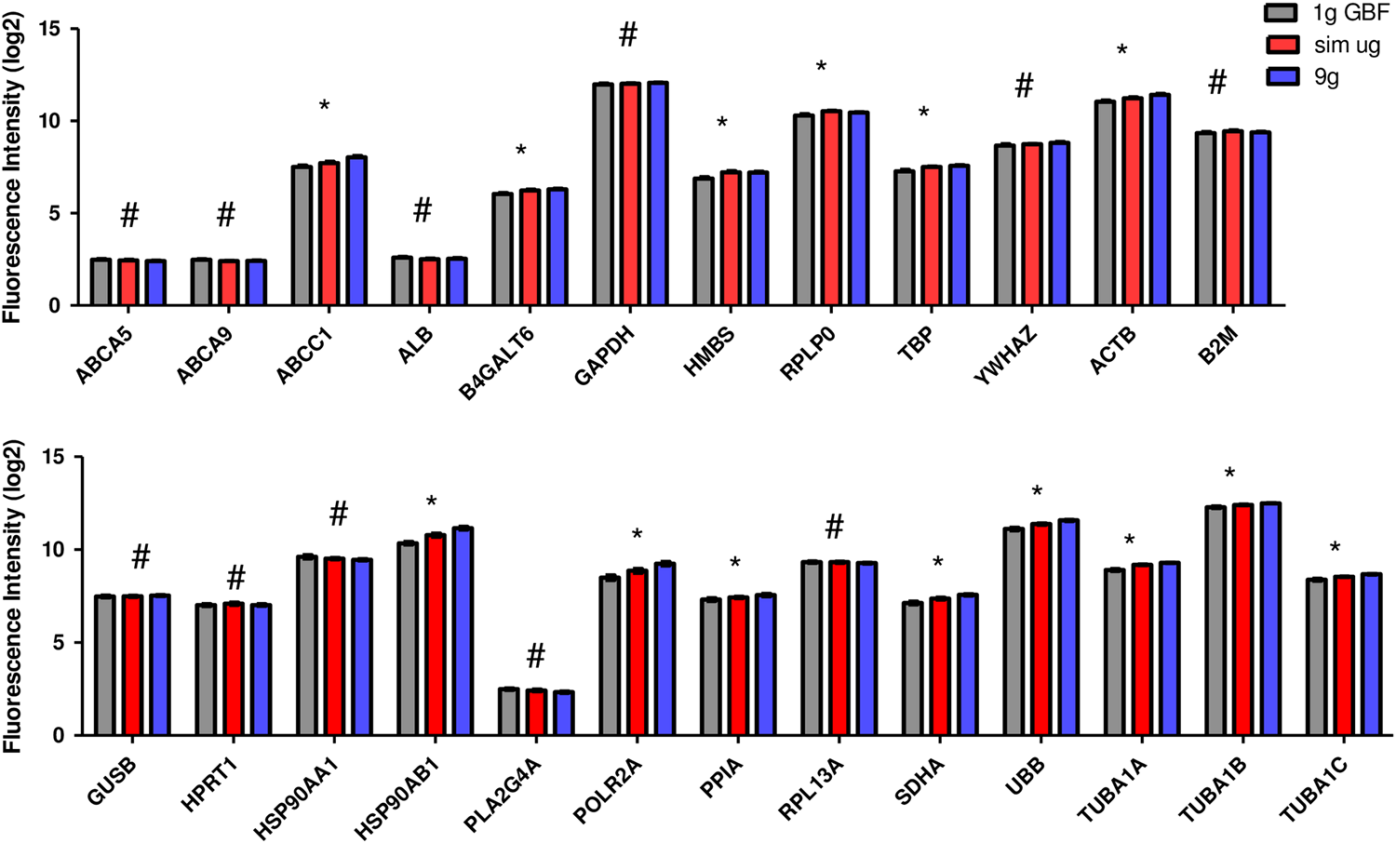
Microarray gene expression analysis of reference genes in samples exposed to different simulated microgravity and hypergravity using ground-based facilities. *Bars* represent mean log2 values of fluorescence intensities with SEM, *n* = a minimum of five samples. *1 g GBF* 1 g ground-based facilities, *sim µg* simulated microgravity. * Significant differences in at least one group comparison, # no significant differences within the three groups according to one-way ANOVA with Tukey’s post-test

In an earlier study based on cells of the innate immune system, we investigated reference genes commonly used for expression analysis (Table 2) and their expression pattern in altered gravity. The microarray data collected for U937 cells during the 19th DLR PFC and the TEXUS-49 mission were screened for the reference genes listed in Table 2. In addition, some evolutionarily highly conserved ABC transporters were tested for their suitability as new reference genes. Table 3 summarizes those genes, which were previously identified to be stably expressed over all analyzed gravity conditions from seconds to minutes and which we suggested as suitable reference genes for platform spanning microgravity research of cells of the monocyte–macrophage system.^22^ We performed the reference gene analysis for the Jurkat T cell expression data set with all the genes listed in Tables 2 and 3. The mean values and standard deviations were calculated from all single expression data from all samples for each gene and platform and the coefficient of variation value was determined (Fig. 4a–c). Typically, mean coefficient of variation values of up to 25% are observed for reference genes.^24^ This criterion was met by all tested genes. In addition, the web-based tool RefFinder^25^ was used to calculate a ranking of the reference genes for each platform. This program can be used for the evaluation and screening of reference genes from extensive datasets. RefFinder^25^ integrates the computational programs geNorm,^26^ Normfinder,^27^ Bestkeeper,^28^ and the comparative delta Ct method.^29^ Based on these four programs, the reference gene candidates are compared and ranked for each individual program. Finally, the four results are summarized into a superior ranking. Subsequently, the RefFinder outputs of the individual platforms for each gene were averaged and a global ranking was calculated (Fig. 4d). According to this ranking, the five most appropriate reference genes were: *ACTB*, *GAPDH*, *TUBA1AB*, *TUBA1A*, and *TUBA1C*. However, our expression data sets also allow a direct comparison of the data obtained for the different gravity conditions. We therefore directly compared expression data obtained for the gravity conditions 1 g in-flight, hypergravity, and microgravity and statistically tested the results with a one-way analysis of variance and Tukey’s post-test to compare multiple groups (Figs. 5–7). The summarized results are displayed in Tables 4 and 5. We could identify in total five genes in Jurkat T cells that are stably expressed over all tested gravity conditions in all platforms: *ABCA5, GAPDH, HPRT1, PLA2G4A,* and *RPL13A*. Two of these genes, namely *ABCA5* and *GAPDH* showed also stable gene expression in the formerly tested U937 cells. We could therefore identify two genes that could serve as reference genes equally effective for cells of the innate and the adaptive immune system under 1 g and altered gravity conditions for the platforms parabolic flight, sounding rockets and GBFs. However, the comparison of the original data with the ranking calculated by the RefFinder web-based tool shows some discrepancies.

**Table 2:** Commonly used reference genes for gene expression analyses (more than one accession number is displayed in case different transcripts exist)

Gene	Gene symbol	NCBI GenBank accession number
β-Actin	ACTB	NM_001101
Albumin	ALB	NM_000477
β-2 Microglobulin	B2M	NM_004048
Beta-1,4-galactosyltransferase 6	B4GALT6	NM_004775
Glyceraldehyde-3-phosphate dehydrogenase	GAPDH	NM_002046
Glucoronidase beta	GUSB	NM_000181.3, NM_001284290.1; NM_001293104.1; NM_001293105.1; NR_120531.1
Hydroxymethylbilane synthase	HMBS	NM_000190; NM_001024382
Hypoxantine phosphoribosyltransferase 1	HPRT1	NM_000194
Heat shock protein 90 kDa	HSP90AA1	NM_001017963; NM_005348; NM_001017963; NM_005348; NM_005348; NM_001017963
Heat shock protein 90 kDa	HSP90AB1	NM_007355
Phospholipase A2	PLA2G4A	NM_024420
RNA polymerase II	POLR2A	NM_000937
Peptidylprolyl isomerase A	PPIA	NM_021130
Ribosomal protein L13	RPL13A	NM_012423
Ribosomal protein lateral stalk subunit P0	RPLP0	NM_001002; NM_053275
Succinate dehydrogenase complex flavoprotein subunit A	SDHA	NM_004168
TATA box binding protein	TBP	NM_001002; NM_053275
α-Tubulin	TUBA1	NM_006000 (TUBA4A alias TUBA1),
Ubiquitin C	UBC	NM_021009
Tyrosine 3-monooxygenase/tryptophan 5-monooxygenase activation protein Zeta	YWHAZ	NM_001135699; NM_001135700; NM_001135701; NM_001135702; NM_003406; NM_145690

**Table 3:** Description of reference genes found to be stable under conditions of altered gravity^22^ (more than one accession number is displayed in case different transcripts exist)

Gene	NCBI GenBank accession number	Chromosomal location	Function
ALB (albumin)	NM_000477	Chr. 4q13.3	Regulation of blood plasma colloid osmotic pressure, acts as a carrier protein for a wide range of endogenous molecules, has esterase-like activity
B4GALT6 (beta-1,4-galactosyltransferase 6)	NM_004775	Chr. 18q12.1	Type II membrane-bound glycoprotein, galactose transfer to acceptor sugars
GAPDH (glyceraldehyde-3-phosphate dehydrogenase)	NM_002046	Chr. 12p13.31	Carbohydrate metabolism: reversible oxidative phosphorylation of glyceraldehyde-3-phosphate, uracil DNA glycosylase activity in the nucleus, protein contains a peptide with antimicrobial activity
HMBS (hydroxymethylbilane synthase)	NM_000190; NM_001024382	Chr. 11q23.3	Enzyme of the heme biosynthetic pathway, catalyzes the head to tail condensation of four porphobilinogen molecules into the linear hydroxymethylbilane
YWHAZ (tyrosine 3-monooxygenase/tryptophan 5-monooxygenase activation protein Zeta)	NM_001135699; NM_001135700; NM_001135701; NM_001135702; NM_003406; NM_145690	Chr. 8q22.3	Mediates signal transduction by binding to phosphoserine-containing proteins
ABCA5 (ATP-binding cassette subfamily A member 5)	NM_018672; NM_172232	Chr. 17q24.3	ATP-binding cassette (ABC) transporter, transport various molecules across extra- and intracellular membranes
ABCA9 (ATP-binding cassette subfamily A member 9)	NM_080283	Chr. 17q24.2	ATP-binding cassette (ABC) transporter, transport various molecules across extra- and intracellular membranes
ABCC1 (ATP-binding cassette subfamily C member 1)	NM_004996; NM_019862; NM_019898; NM_019899; NM_019900	Chr. 16p13.11	ATP-binding cassette (ABC) transporter, involved in multi-drug resistance, multispecific organic anion transporter

**Table 4:** Gene expression stability analysis in Jurkat T lymphocytes of stable reference genes previously tested in U937

Reference genes that have been previously identified in U937 cells to be stable under altered gravity conditions^22^ were also reviewed in Jurkat T cells. Comparisons of gravity conditions for different real and simulated microgravity platforms are shown on the left side. ABCA5 and GAPDH (bold) were stable in all tested comparisons in Jurkat T cells as well as in previously analyzed U937 cells^22^ *1 g* IF 1 g in-flight, *μg* microgravity, *1 g GBF* 1 g control for clinostat and 9 g centrifuge experiments, *BL* baseline (representing the hypergravity phase), *blue* stable expression, *red* significantly altered expression in the respective comparison, *sim μg* simulated microgravity

**Table 5:** Analysis of further commonly used reference genes for gene expression stability in Jurkat T lymphocytes

Reference genes that have been previously shown to be differentially expressed in U937 cells under altered gravity conditions^22^ were analyzed in Jurkat T cells. Comparisons of gravity conditions for different real and simulated microgravity platforms are shown on the left side. Tubulin-alpha is represented by three transcripts TUB1A, TUB1B, TUB1C. A stable gene expression could be shown over all tested platforms and comparisons for HPRT1, PLA2G4A, and RPL13A (bold) *1 g IF* 1 g in-flight, *μg* microgravity, *1 g GBF* 1 g control for clinostat and 9 g centrifuge experiments, *BL* baseline (representing the hypergravity phase), *blue* stable expression, *red* significantly altered expression in the respective comparison, *sim μg* simulated microgravity

### Identification of stable gene expression in different gravity environments

**Fig. 8:**
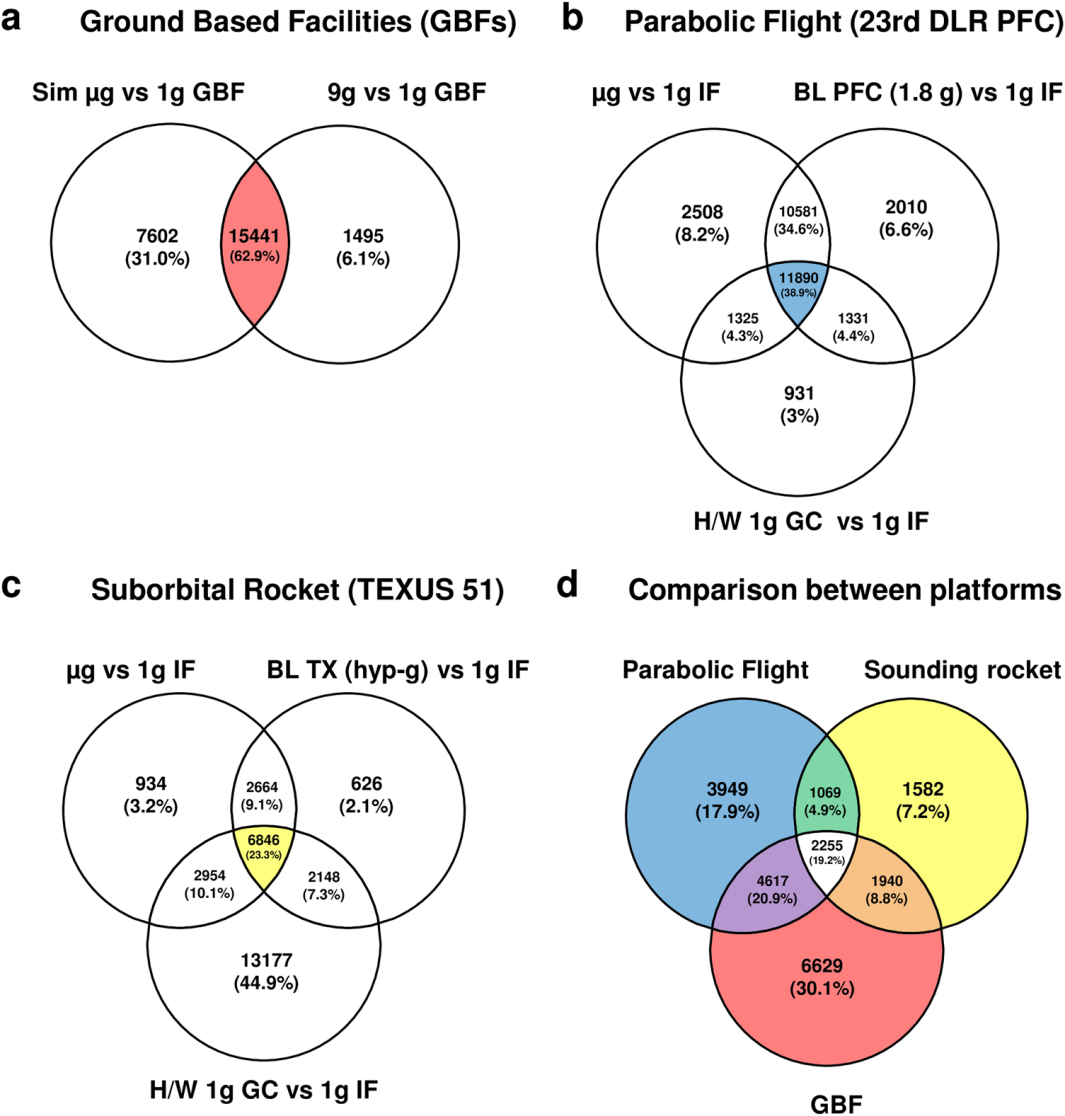
Venn diagram of comparisons of stable gene expression between gravity conditions for the different platforms and determination of intersections of overlapping transcripts. **a** Ground-based facilities (*GBFs*): 15,441 genes are stably expressed for the comparisons simulated µg vs. 1 g GBF and 9 g vs. 1 g GBF. **b** 23rd DLR parabolic flight campaign: 11,890 genes are stably expressed between the comparisons μg vs. 1 g in-flight (*IF*), 1.8 g vs. 1 g IF and 1 g H/W ground control (*GC*). **c** TEXUS-51 sounding rocket campaign: 6846 genes show stable gene expression for the comparisons μg vs. 1 g IF, baseline (*BL*) vs. 1 g IF, and 1 g H/W GC vs. 1 g IF. **d** Intersection of stable gene expression for all analyzed platforms. The comparison of all three platforms results in an intersection value of 2255 stable expressed genes, while when comparing only the real microgravity platforms (parabolic flight and sounding rocket) 3324 genes were stably expressed over all investigated conditions. (criteria for stable gene expression: fold change in the range of ±1.1 and *p*-value >0.05)

We further analyzed our complex data sets to identify additional genes and cellular and molecular processes and pathways that remain stable in an altered gravity environment from seconds to minutes. The expression profiles of the individual platforms were explicitly screened for genes with stable expression values. Comparisons between groups within a platform were performed based on the search criterion of a low-fold change (FC) within the range of ±1.1 and a *p*-value of >0.05. This cutoff value was chosen because other studies have shown that also slightly differentially expressed genes with a FC of approximately ±1.2 played a significant role in the downregulation of oxidative phosphorylation pathway in diabetes.^30–32^ In order to unambiguously identify the stable expression of genes, we therefore decided to search for transcripts with a FC in the range of ±1.1 in this study. The expression data of the individual groups were compared within each platform and all transcripts, which met the criterion −1.1≤FC≤+1.1 were identified. Subsequently, the intersection of the transcripts was determined, which met the criterion for all group comparisons. In the GBFs experiments, 15,441 genes were identified which showed a FC value of ±1.1 in both, the simulated microgravity vs. 1 g and the comparison 9 g centrifugation vs. 1 g (Fig. 8a). This corresponds to 22.9% of all transcripts located on the microarray. In the parabolic flight data set, comparisons of 0 vs. 1 g in-flight, 1.8 vs. 1 g in-flight, and 1 g hardware ground control vs. 1 g in-flight were performed. A total of 11,890 genes were identified, which showed a FC of ±1.1 in all three comparisons (Fig. 8b). This corresponds to 17.6% of all transcripts located on the microarray. For the experiments performed on the third microgravity platform (suborbital ballistic rocket), the following group comparisons were made: μg vs. 1 g in-flight, baseline vs. 1 g in-flight, and 1 g hardware ground control vs. 1 g in-flight. About 6846 genes representing 10.1% of all microarray transcripts exist, which show an FC value of ±1.1 for the intersection (Fig. 8c). In summary, between 10.1 (suborbital ballistic rocket) and 22.9% (2D clinostat and centrifuges) of all transcripts remained completely stable during any gravity condition between 10^−4^ and 9 g. Furthermore, in a cross-platform analysis, we searched for all genes with a FC of ±1.1 in the GBFs, parabolic flight, and sounding rocket intersection data. We identified 2255 that met these criteria (Fig. 8d), representing 3.3% of all transcripts. Further analysis comparing only the real microgravity platforms showed that 3324 genes (4.9% of all transcripts) fulfilled these requirements (Fig. 8d).

After analysis of the microarray data for identification of robust non-changing transcripts with a FC threshold of ±1.1, we found 26,304 = 39.9% non-changing genes after 20 s and 13,398 = 19.8% non-changing genes after 5 min of microgravity compared to 1 g in-flight controls. After selection for changing transcripts with a FC threshold of ±1.3, we found 279 = 0.4% altered transcripts after 20 s and 1873 = 2.7% altered transcripts after 5 min microgravity. The number of transcripts in the range of minimal changes (threshold ±1.1−1.3) were 40,945 = 60.6% after 20 s microgravity, and 52,257 = 77.3% after 5 min microgravity.

In conclusion, 97.3–99.6% of all transcripts were not or only minimally altered in microgravity (threshold ±1.3), while 19.8–39.9% were identified as stably expressed (threshold ±1.1).

### Higher stability of gene transcription in 2D clinostat experiments compared to real microgravity

Parallel transcriptome investigation after 5 min simulated vector-averaged microgravity in 2D clinostat revealed 23,043 non-changing genes (34.1% of all transcripts) in contrast to 5 min real microgravity in the TEXUS suborbital ballistic rocket experiment exhibiting 13,398 non-changing genes (19.8% of all transcripts). Both sample groups were compared to their respective internal 1 g controls. Because only 2774 additional non-changing genes (4.1%) were detected after the preceding launch and hypergravity phase of TEXUS, at least 6873 microgravity-responsive genes could not be detected in clinostat experiments. Therefore, 2D clinostat experiments are not sufficient to identify the full spectrum of microgravity-responsive genes. Due to technical and operational limitations of the 2D clinostat, it was not possible to perform reliable 2D clinostat experiments with 20 s simulated microgravity for a comparison with the parabolic flight experiments.

### Olfactory and taste receptor gene expression is particularly robust to altered gravity

Transcripts which were stably expressed in all gravity conditions were further analyzed and data were searched for genes with an existing annotation. For the triple platform comparison between ground-based experiments (2D clinostat and centrifuge), parabolic flight, and suborbital ballistic rocket flight, 1540 out of 2255 transcripts could be identified. For the comparison between the real microgravity platforms parabolic flight and sounding rocket, 2612 annotated genes were identified from 3324 transcripts. For the filtered stably expressed genes, a gene set enrichment analysis was performed using the program DAVID 6.8 and pathway databases KEGG and gene ontology were searched. The more genes could be assigned to the same pathway, the more significant turned out the results. In a detailed downstream analysis, similar pathways were grouped into clusters and a total of three significant gene clusters were identified in the two data sets, representing the functional topics G protein-coupled receptors (GPCR) signaling, zinc finger-involved DNA binding, and bacterial defense.

**Fig. 9:**
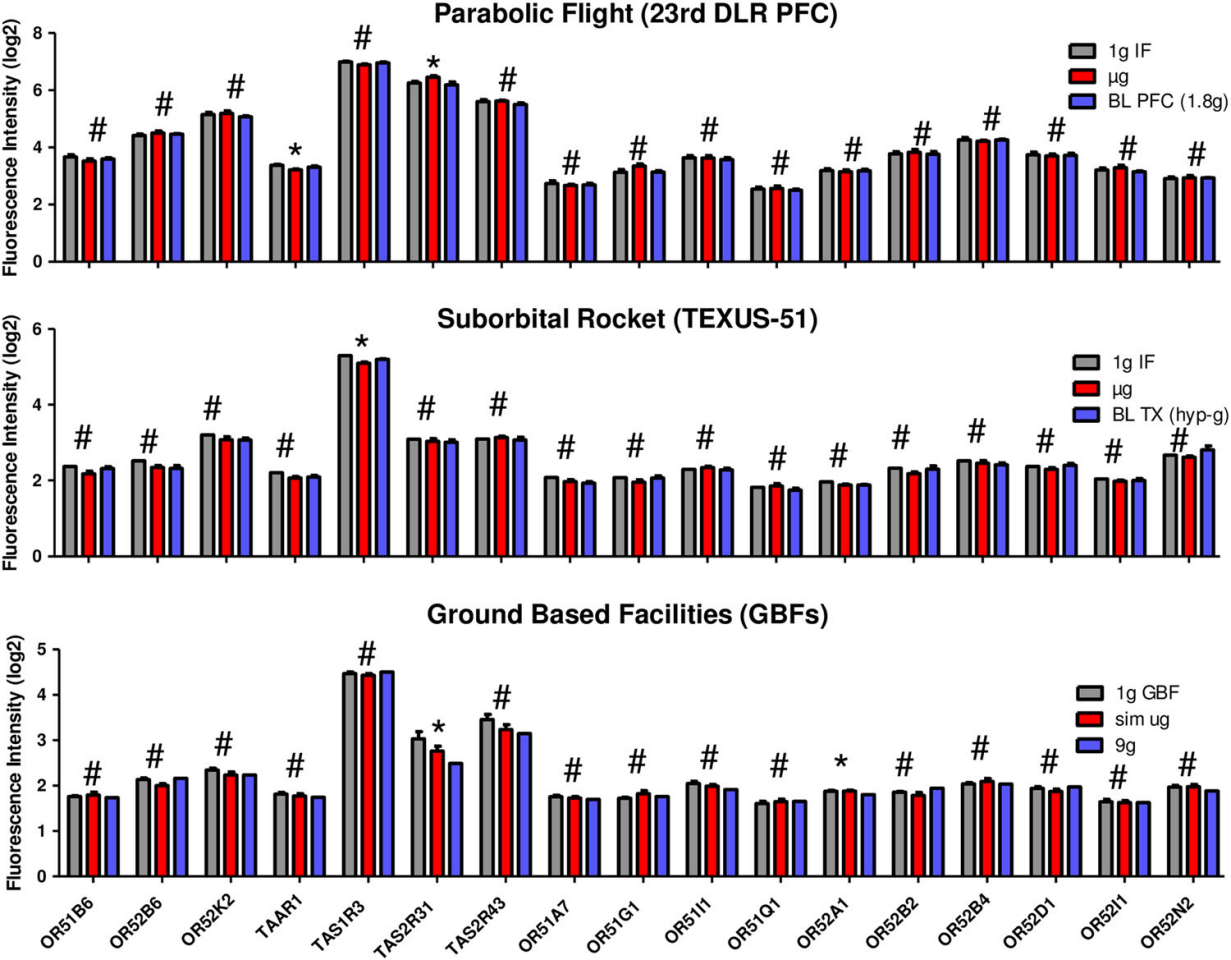
Microarray gene expression analysis of olfactory and taste receptor genes in samples exposed to different gravity conditions during parabolic flights, sounding rockets, or ground-based facilities. *Bars* represent mean log2 values of fluorescence intensities with SEM, *n* > 5. * Significant differences in at least one group comparison, # no significant differences within the three groups according to one-way ANOVA with Tukey’s post-test

**Fig. 10:**
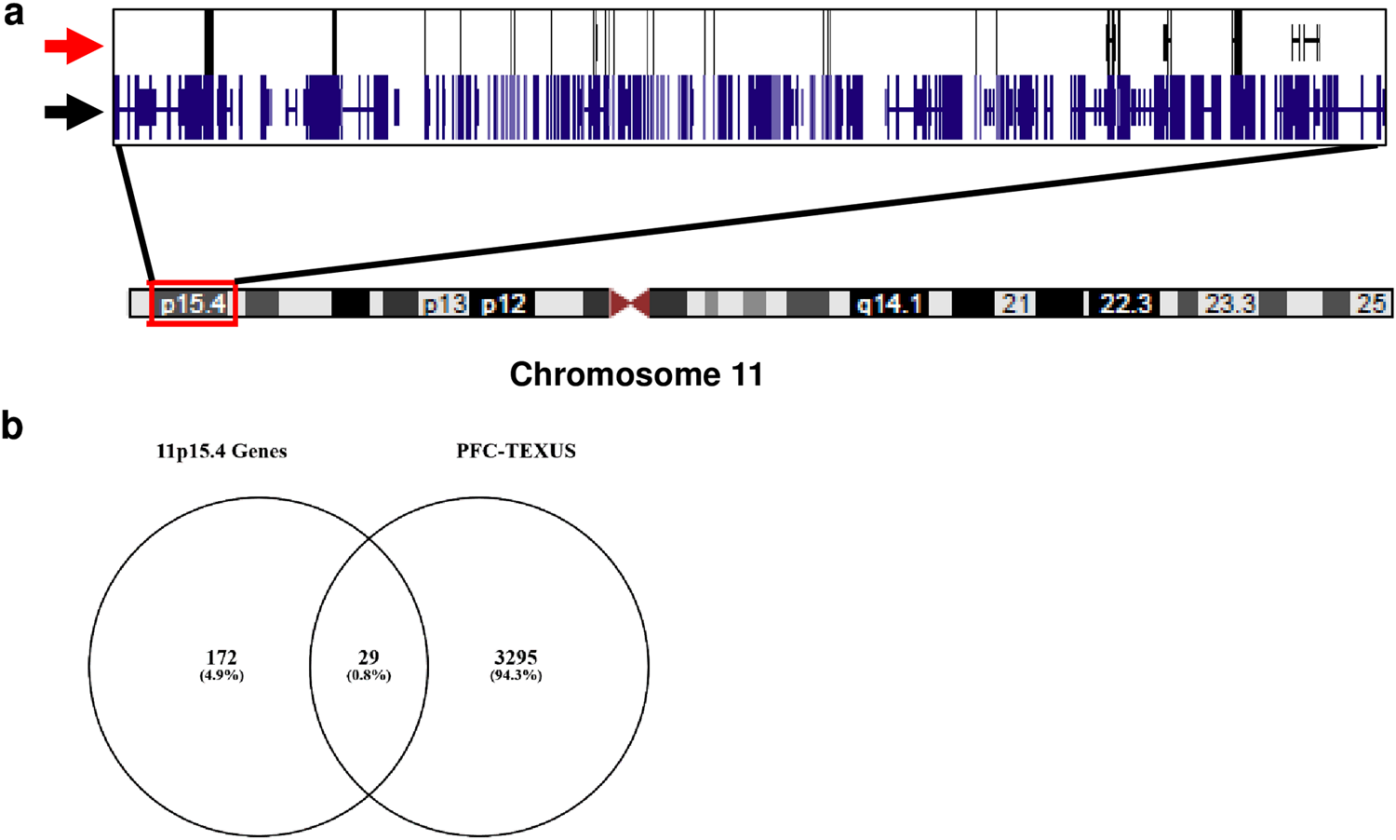
**a** Schematic of human chromosome 11. The chromosomal region 11p15.4 is highlighted by the *red box*. A zoom-in is displayed with all annotated genes located in the chromosomal band marked by the *black arrow* and the genes identified to be stably expressed marked by the *red arrow*. The stably expressed genes show a homogeneous distribution. (Chromosomal locations were displayed with the UCSC Genome Browser.^67^) **b** Venn diagram of comparisons of stable gene expression between all gravity conditions (the parabolic flight and suborbital ballistic rocket experiment) and gene expression on chromosomal region 11p15.4

GOrilla gene cluster analysis (Supplementary Note) revealed an over-representation of GPCR signaling and sensory perception genes. The appearance of olfactory receptor activity and sensory perception of smell, additionally to the G protein-coupled receptor activity and signaling, seems to be surprising at first glance. However, other groups already reported the expression of olfactory and taste receptors in a variety of cells of the immune system.^33^ We chose seven genes, *TAS1R3*, *TAS2R31*, *TAS2R43*, *TAAR1*, *OR51B6*, *OR52B6*, *OR52K2*, described to be abundantly expressed in T cells and screened our expression data sets. Additionally, we compared the results of our gene enrichment analysis with the expression profile published by Malki et al.^33^ and found a match for 10 further olfactory receptors. Table 6 lists the investigated olfactory and taste receptor genes. We searched our expression data sets again for these 17 genes and calculated for each gene and gravity condition the mean and SEM values and ran a statistical one-way ANOVA with Tukey’s multiple groups post-test (Fig. 9). The results are summarized in Table 7. Interestingly, 14 of 17 genes show a stable gene expression in all comparisons and they all belong to the gene classes OR1 and OR2 located on chromosomal region 11p15.4 (Fig. 10a). One taste receptor, TAS2R43, was also found to be stably expressed under all gravity conditions. However, the gene for this receptor is located on chromosome 12p13.2. While 3324 genes = 4.9% of all transcripts were stably expressed (FC in the range of ±1.1 and *p*-value >0.05) in all investigated gravity environments during parabolic flight and suborbital ballistic rocket experiments, the share of stable genes was 14.4% = 3-fold higher in chromosomal region 11p15.4 (Fig. 10b). Therefore, chromosomal region 11p15.4 can be considered as a region of stability for gene expression in altered gravity.

**Table 6:** GenBank accession number and chromosomal location of olfactory and taste receptor genes expressed in cells of the immune system

Gene	NCBI GenBank accession number	Chromosomal location
OR1A7	NM_001004749	Chr. 11p15.4
OR51B6	NM_001004750	Chr. 11p15.4
OR51G1	NM_001005237	Chr. 11p15.4
OR51I1	NM_001005288	Chr. 11p15.4
OR51Q1	NM_001004757	Chr. 11p15.4
OR52A1	NM_012375	Chr. 11p15.4
OR52B2	NM_001004052	Chr. 11p15.4
OR52B4	NM_001005161	Chr. 11p15.4
OR52B6	NM_001005162	Chr. 11p15.4
OR52D1	NM_001005163	Chr. 11p15.4
OR52I1	NM_001005169	Chr. 11p15.4
OR52K2	NM_001005172	Chr. 11p15.4
OR52N2	NM_001005174	Chr. 11p15.4
TAAR1	NM_138327	Chr. 6q23.2
TAS1R3	NM_152228	Chr. 1p36.33
TAS2R31	NM_176885	Chr. 12p13.2
TAS2R43	NM_176884	Chr. 12p13.2

**Table 7:** Analysis of olfactory and taste receptor genes for expression stability in Jurkat T lymphocytes under altered gravity conditions

About 17 different olfactory and taste receptor genes previously reported to be expressed in T cells have been analyzed for their expression stability under altered gravity conditions. With the exception of TAAR1, TAS1R3, and TAS2R31, all investigated genes (bold) were stably expressed in each altered gravity condition in Jurkat T cells *1 g IF* 1 g in-flight, *μg* microgravity, *1 g GBF* 1 g control for clinostat and 9 g centrifuge experiments, *BL* baseline (representing the hypergravity phase), *sim μg* simulated microgravity

## Discussion

In our study, we investigated the stability of gene expression response in non-activated human T lymphocytic cells in 20 s and 5 min microgravity and in hypergravity. We revealed an overall high stability of gene expression in microgravity, with olfactory gene expression being particularly robust to altered gravity. We could identify five genes in Jurkat T cells out of the entire transcriptome that were stably expressed in all tested gravity conditions and in all platforms: *ABCA5, GAPDH, HPRT1, PLA2G4A,* and *RPL13A*. Two of these transcripts were also known to be stably expressed in U937 cells in all gravity conditions:^22^ *ABCA5* and *GAPDH*. We therefore propose *ABCA5* and *GAPDH* as reference genes in immune cells for experiments in altered gravity.

In a first approach, we revised the results from an earlier study with myelomonocytic cells^22^ and searched for the stable expression of reference genes in the data sets obtained from the 23rd PFC, the TEXUS-51 sounding rocket mission, and 2D clinorotation and centrifugation experiments in GBFs. Two out of eight genes stably expressed under altered gravity conditions in monocytic U937 cells^22^ could also be identified to be unaltered in Jurkat T cells. The first one was *GAPDH*, which has multiple known functions in several localizations within a cell. The best characterized and longest known function of GAPDH occurs in the cytoplasm, as a key enzyme in glycolysis catalyzing the first step of the pathway by converting d-glyceraldehyde-3-phosphate (G3P) into 3-phospho-d-glyceroyl phosphate in the presence of NAD^+^ and phosphoric acid. Nuclear tasks of GAPDH comprise transcription, RNA transport, DNA replication, and apoptosis, and are probably conferred by the nitrosylase activity that mediates cysteine S-nitrosylation of nuclear target proteins.^34^ GAPDH also modulates the organization and assembly of the cytoskeleton by facilitating CHP1-dependent microtubule and membrane associations.^35^ GAPDH expressed on the cell surface is able to internalize iron in a transferrin-mediated way.^36, 37^ Furthermore, GAPDH is a component of the GAIT (gamma interferon-activated inhibitor of translation) complex which mediates interferon-gamma-induced transcript-selective translation inhibition in inflammation processes.^38^ And GAPDH was found to interact with the TNF receptor-associated factor 2 (TRAF2), a protein required for TNF-alpha-mediated NF-κB activation.^39^ This is only a brief list of all discovered functions of GAPDH so far. It becomes clear that this enzyme is involved in many fundamental cell processes making it obviously a “housekeeping” gene. This is probably also the reason why it is highly conserved throughout evolution. GAPDH is found in all eukaryotes as animals, plants, and fungi, as well as in bacteria and archaea. In that light, it is easily conceivable that cells have come up with a robust mechanism of stable GAPDH expression even under extreme conditions like altered gravity.

The second gene that we identified to be stable under short-term and mid-term altered gravity conditions in U937 and Jurkat T cells is a membrane-associated protein encoded by the *ABCA5* gene. It is a member of the ATP-binding cassette (ABC) transporter superfamily. In general, ABC transporters mediate the shuttling of various molecules across extra- and intracellular membranes. *ABC* genes are further divided into seven subfamilies being ABC1, MDR/TAP, MRP, ALD, OABP, GCN20, and white. ABCA5 belongs to the ABC1 subfamily, which is found exclusively in multicellular eukaryotes. Unfortunately, the substrate and the exact function of ABCA5 are not known. However, it has lately been suggested as a cholesterol transporter in the brain, connected to amyloid-β peptide generation and Alzheimer’s disease,^40^ and has been associated with the progression of acute myeloid leukemia.^41^ The tight regulation of transporting substances across membranes ensures survival under challenging conditions and thus facilitates adaptation to new environments. Therefore, it seems rather not surprising to find members of the ABC1 subfamily stably expressed under altered gravity, which ultimately may be a new environmental condition that cells might have to adapt to.

Moreover, we were able to identify three of the commonly applied reference genes as stably expressed under altered gravity (Table 2): (1) Hypoxanthine-guanine phosphoribosyltransferase (HGPRT, gene name *HPRT1*) converts guanine to guanosine monophosphate and hypoxanthine to inosine monophosphate. It transfers the 5-phosphoribosyl group from 5-phosphoribosylpyrophosphate onto the purine. Thus, HGPRT plays a central role in the generation of purine nucleotides through the purine salvage pathway. (2) Cytosolic phospholipase A2 (gene name *PLA2G4A*) plays a key role in the production of the precursors of eicosanoids and platelet-activating factor by selectively hydrolyzing arachidonyl phospholipids releasing arachidonic acid and exerting a lysophospholipid activity. It is therefore implicated in the initiation of the inflammatory response. (3) Ribosomal protein L13a (*RPL13A*) is a very widely used reference gene for expression studies in various cell and tissue types. Human RPL13a was shown to be dispensable for canonical ribosome function but indispensable for efficient ribosomal RNA methylation, hypothesizing that L13a may have evolved from an essential ribosomal protein in lower eukaryotes to having a role as a dispensable extra-ribosomal function in higher eukaryotes.^42^ Like *GAPDH* and *ABCA5*, these three genes are involved in basic cellular mechanisms and are indispensable for cellular functions. Taken together, we were able to identify in total five commonly used reference genes to be stably expressed under all tested gravity conditions in human Jurkat T lymphocytes. Furthermore, we could show that two of these genes, *GAPDH* and *ABCA5*, which are evolutionarily highly conserved were indeed stable for all tested conditions in two cell types representative for immune cells of the innate and adaptive system and can therefore be regarded as general immune cell reference genes.

In a second approach, we applied an automatized analysis of all expression data from all platforms in Jurkat T cells. The microarray expression data sets were screened for stable genes with a FC of ±1.1 and a *p*-value >0.05. Downstream gene annotation enrichment analysis were performed with DAVID 6.8 and GOrilla for positively screened genes. We found a significant enrichment of pathways involved in detection of chemical stimulus in GPCR signaling, sensory perception of smell and olfactory receptor activity in GOrilla pathway analysis (http://cbl-gorilla.cs.technion.ac.il). We therefore investigated the expression of several olfactory and taste receptors, and found a surprising stability throughout altered gravity conditions. The OR51 and OR52 subfamilies belonging to the Class I odorant receptor genes, previously described to be expressed in cells of the immune system,^33^ are stably expressed in the comparisons microgravity vs. 1 g and hypergravity vs. 1 g (Tables 6, 7). Interestingly, these two olfactory receptor subfamilies are all located on chromosome 11p15.4.

In our study, we were able to detect a new class of reference genes, the olfactory receptors, displaying a very high stability of expression in altered gravity over different time periods and platforms and we recommend to consider this class of reference genes for space-related expression analysis. Olfactory receptors are members of the GPCRs family, which are potential mechanosensors. A more detailed analysis of gene expression in the chromosomal region 11p15.4 revealed that from totally 201 genes, 29 genes (14.4%) were stably expressed, which was three-fold more than in the average of the entire genome, where 3324 genes = 4.9% of all transcripts were stably expressed in all gravity conditions. We determined the distribution of the genes and found a relative homogeneous arrangement throughout this chromosomal part. We therefore hypothesize that the reason for this region of stability of gene expression stability in 11p15.4 could be based on a very stable chromatin structure that stays unaltered under all tested gravity conditions (Fig. 10a).

Expression of olfactory genes in a lymphocyte cell line seems remarkable, however it is a changing paradigm that proteins and enzymes only exert one function within a cell. There is growing acceptance that proteins may play multiple roles within a cell, like for instance GAPDH. In the past, there has been evidence of an ectopic expression of chemosensory receptors in a variety of peripheral tissues, and it was demonstrated that different types of isolated human blood leukocytes functionally express members of an olfactory receptor family, the TAARs, which detect certain biogenic amines with high sensitivity.^43^ Recently, also the expression of other chemosensory receptors, such as Class I odorant receptors (ORs), bitter-taste receptors (TAS2Rs), and sweet- and umami-taste receptors (TAS1Rs) in different types of blood leukocytes was found, and it was hypothesized that foodborne flavor chemicals represent receptor-specific bioactives for immune cells guiding chemotactic migration.^33^ Why immune cells respond to food ingredients still remains unclear. However, this ability seems to be fundamental, since it is tightly regulated even under altered gravity conditions. Due to the fact that olfactory and taste receptors expression has been detected immunocytochemically in 40–60% of PMN and T or B cells^33^ and their supposed function in immune cell migration,^33, 44^ it seems unlikely that their stability can be attributed mostly to the absence of function. In contrast, olfactory receptors are members of the GPCRs family, which are potential mechanosensors. Indeed, olfactory receptors have been identified recently as mechanoreceptors^45^ and its ectopic expressions could therefore be considered as a mechanism to confer mechanosensitivity to olfactory receptor carrying cells. Therefore we assume that olfactory receptor genes are genes of mechanosensitive receptors, which are stably expressed under different force conditions.

The experiments of this study were performed in different microgravity environments (10^−2^–10^−3^ g for parabolic flight experiments and 10^−4^ g for TEXUS experiments). Currently, it is not known, how far biological reactions and gene expression are influenced by different levels of low gravity. A 2D clinostat study with 1F6 melanoma cells reported differences in guanylyl cyclase A messenger RNA expression in the range between 0.012 and 0.036 g.^46^ In an ISS BIOLAB experiment measuring the oxidative burst reaction in NR8383 macrophages, indications for a gravitational threshold between 0.3 and 0.5 g were found, whereas it seems that the immediate decline of the oxidative burst reaction was not different between the 10^−2^ and 10^−3^ g for parabolic flight experiments^47^ and the 10^−5^ g for the ISS experiment.^48^ Therefore, the current knowledge provides no clear answer about cellular or molecular thresholds of low-gravity effects or the existence or absence of a correlation between the gravitational force and the biological effect below 1 g.

We found that a high number of genes were stably expressed in all comparisons between microgravity, 1 g, and hypergravity, and even in the 1 g hardware control vs. 1 g in-flight control samples. In our GBF, parabolic flights and suborbital rocket experiments, 22.9, 17.6, and 10.1% of all 67,528 analyzed transcripts were stably expressed in all conditions, respectively (Fig. 4). Analysis for robust non-changing transcripts in microgravity compared to 1 g in-flight controls revealed 39.9% non-changing genes after 20 s and 19.8% non-changing genes after 5 min, while the number of minimal changing genes were 60.6% after 20 s microgravity and 77.3% after 5 min microgravity. Analysis for robust changing transcripts confirmed 0.41% and 2.77% altered transcripts, respectively. In conclusion, about 10–20% of all transcripts remained totally unchanged in any gravitational environment tested (between 10^−4^ and 9 g), about 20–40% remained unchanged in microgravity (between 10^−4^ and 10^−2^ g), and about 97–99% were not significantly altered in microgravity, if strict exclusion criteria were applied. This was the same range as in a study using Jurkat T cells during a space shuttle mission, which demonstrated that 98% of the gene transcripts were not altered.^14^ After 5 min microgravity, 717 annotated transcripts changed between 1.1- and 1.3-fold (*p*-value ≤0.05) and only 54 annotated transcripts between 1.3- and 1.5-fold (*p*-value ≤0.05). The absolute minimum and maximum values for the differentially expressed transcripts were −1.59 and +1.85, respectively. Therefore, we detected no drastic changes of gene expression within 5 min microgravity. In Table 8, we compared the general gene expression response pattern in comparison with other stressors such as chemicals, radiation, heat, infection, and nutrition.^49–61^ Although these studies have usually applied higher threshold criteria for changing gene expression (upto 4.0), the microgravity environment still caused an overall gene expression response far below the gene expression changes upon severe stress induced by glucocorticoids or radiation, where changes in upto 21.46% and 15.79% of the transcripts were observed, respectively. However, these comparisons across different stressors have to be considered with caution and can only provide a simple and general overview, because the assembled studies have been carried out under different conditions with different study objects, types of microarrays, different incubation times, and different FC thresholds. Most importantly, only functional studies will provide answers about the impact and role of gene expression patterns, adaptation, and homeostasis mechanisms in the same way as the first cellular effect of microgravity was discovered more than 30 years ago.^6^ Nevertheless, we obtain a first impression about the magnitude of gene expression effects in a microgravity environment compared to different types of stress.

**Table 8:** Influence of different types of stress on cellular- or tissue-specific gene expression^49–61^

Stressor	Condition/ dose	Exposure time	Organisms/ tissues/cells	Microarray	Number of sample sets	Fold change ±	Differentially expressed transcripts	Differentially expressed transcripts in %	Reference
Altered gravity	Microgravity	20 s	Jurkat T cells	Affymetrix	67,528	1.3	279	0.41%	This study
Altered gravity	Microgravity	5 min	Jurkat T cells	Affymetrix	67,528	1.3	1873	2.77%	This study
Dengue virus infection	NA	48 h	HUVEC	Affymetrix human genome HG-U133A	22,283	4	395	1.77%	49, 53
Deoxynivalenol	5–25 mg/kg	3–24 h	Thymuses, C57Bl6 mice	4 × 44K whole-genome mouse oligo microarrays G4122F, Agilent	39,430	1.5	0–1707	0–4.33%	50, 54
Deoxynivalenol	0.25–0.5 µM	3–24 h	Jurkat T cells	4 × 44K human whole-genome Agilent microarray	27,958	1.5	24–578	0.09–2.07%	51, 55
Deoxynivalenol	2–4 µM	6–24 h	PBMC	4 × 44K human whole-genome Agilent microarray	27,958	1.5	487–938	1.74–3.36 %	52, 56
Giardia lamblia incubation	NA	1.5–18 h	Caco-2 cells	Affymetrix GeneChips Human Genome Focus Array	8746	2	24–214	0.27–2.45 %	52, 56
Heat shock	42 °C	1 h	Human peripheral lymphocytes	NA	4224	2.5	76	1.80%	53, 57
Exercise	NA	30 min exercise	PBMC human	Affymetrix HU133A GeneChip	>47,000	No threshold	311	0.66%	54, 58
Exercise	NA	After 60 min post-exercise	PBMC human	Affymetrix HU133A GeneChip	>47,000	No threshold	552	1.17%	54, 58
Tobacco smoke	NA	5 min exposure to smoke	PBMC human	NA	30,000	No threshold	153	0.51%	55, 59
Western style diet	NA	3 months	C57Bl/6J mice	Sentrix MouseRef-8 v1 24K Expression BeadChips (Illumina)	25,600	1.2	248	0.97%	56, 60
Western style diet	NA	6 months	C57Bl/6J mice	Sentrix MouseRef-8 v1 24K Expression BeadChips (Illumina)	25,600	1.2	121	0.47%	56, 60
Glucocorticoid stimulated with dexamethasone	10^7^ M	18 h	PBMCs	GEM microarray Incyte, St. Louis, MO	9182	1.4	1970	21.46%	57, 61
Hypoxia	1% O_2_	16 h	Primary human monocytes	Affymetrix Human Genome-U133A 2.0 GeneChips	22,283	1.5	1213	5.44%	58, 62
Ionizing radiation	5 Gy	4 h post irradiation	Human lymphoblastoid cell lines	Affymetrix U95A_v2 GeneChip microarray	12,625	1.3	526	4.17%	59, 63
Ultraviolet radiation	10 J/m^2^	24 h post UV exposure	Human lymphoblastoid cell lines	Affymetrix U95A_v2 GeneChip microarray	12,625	1.3	1113	8.82%	59, 63
Neutron radiation	0.25 Gy	Day 1–7 post-irradiation	Mouse blood	Agilent Mouse Gene Expression 4 × 44K v2 Microarray Kit (G4846A)	39,430	1.5	46–691	0.12–1.75%	60, 64
Neutron radiation	1 Gy	Day 1–7 post irradiation	Mouse blood	Agilent Mouse Gene Expression 4 × 44K v2 Microarray Kit (G4846A)	39,430	1.5	1956–6226	4.96–15.79%	60, 64
X-ray radiation	1 Gy	Day 1–7 post irradiation	Mouse blood	Agilent Mouse Gene Expression 4 × 44K v2 Microarray Kit (G4846A)	39,430	1.5	563–765	1.43–1.94%	60, 64
X-ray radiation	4 Gy	Day 1–7 post irradiation	Mouse blood	Agilent Mouse Gene Expression 4 × 44K v2 Microarray Kit (G4846A)	39,430	1.5	1585–3577	4.02–9.07%	60, 64
Radiation (60 Co)	10 Gy / 5 × 2 Gy	24 h post irradiation	MCF7 breast carcinoma	Custom made #	7680	1.5	1229/1049	16.00/13.67%	61, 65
Radiation (60 Co)	10 Gy / 5 × 2 Gy	24 h post irradiation	DU145 prostate carcinoma	Custom made #	7680	1.5	614/881	7.99/11.47%	61, 65
Radiation (60 Co)	10 Gy / 5 × 2 Gy	24 h post irradiation	SF539 gliosarcoma	Custom made #	7680	1.5	611/1162	7.96/5.13%	61, 65

*NA* not applicable, *no threshold* no threshold is stated for fold change, i.e., all significant differentially expressed genes were considered for the analysis

#Incyte UniGEM2 set (Fremont, CA) and Research Genetics Named Genes set (Huntsville, AL)

Therefore, we suppose a high stability of gene expression in microgravity and that altered transcripts are mostly involved in fast cellular adaptation processes, which can occur in principle very fast after exposure to microgravity.^48^ We assume that microgravity alters gene expression homeostasis not stronger than other environmental factors and does not impose an unacceptable risk during long-term space missions.

## Methods

### Cell culture

Human Jurkat T cells (ATCC Clone E6-1, TIB152)^23^ were cultivated under different gravity conditions in RPMI 1640 medium (Biochrom/Merck Millipore, Germany), supplemented with 10% fetal bovine serum (FBS Superior; Biochrom/Merck Millipore, Germany), 2 mM glutamine (low endotoxin; Biochrom, Germany), and 100 U/ml penicillin, as well as 100 µg/ml streptomycin (Biochrom, Germany). Cells were seeded with a density of 0.2 × 10^6^ cells/ml and the medium was changed every 48 h.

### Parabolic flight experiment platform

Parabolic flights are an ideal platform to study initial and primary effects in mammalian cells and the associated rapid responsive molecular alterations excluding influences and interferences of secondary signal cascades. Parabolic flights offer a sequence of consecutive gravity conditions including 1, 1.8 g, and microgravity (µg) with a quality of 10^−2^–10^−3^ g. We designed and constructed an experimental system, which allows cell culture experiments during parabolic flights on board the Airbus A300 ZERO-G (reg. no. F-BUAD), which has been used already for different parabolic flight experiments. Primary importance was placed on realizing the direct safety technique during the development activity. The experimental structure consists of three experiment racks (storage rack for cell culture containers before the experiments at 36.5 °C, cooling rack for storage of cell culture containers after cell lysis at 4 °C, and a working rack for handling and execution of the experiments). The modular system is able to accommodate up to 54 cell culture containers (double containment) for each flight and allows storage of cell cultures until the start of the experiment, injection of a fluid (culture medium) at any defined time during the parabolic maneuver, and automatic injection of a second fluid (lysis buffer) after 20 s at the end of a defined gravity phase. Appropriate in-flight controls were obtained during the 1 g flight phase directly before the parabola. Injection of all fluids operates automatically and is pre-programmed, while exchange of cell culture containers and supervision of the experiment was performed manually. During the 23rd DLR PFC, we investigated the gene expression in Jurkat T cells in microgravity and hypergravity (1.8 g) compared to in-flight 1 g. Experiments were only conducted during the first parabola to assure that detected differential gene expression levels were a result of the effect of gravitational change and not an accumulated long-term effect (Fig. 1).

### Preparation and execution of the parabolic flight experiments

During the 23rd DLR PFC, 1 × 10^7^ Jurkat T cells in 10 ml medium (RPMI 1640 supplemented with 100 U/ml penicillin, 100 µg/ml streptomycin, 2 mM glutamine and 10% FBS) were filled into 200 ml Nutrimix bags (B. Braun Melsungen, Germany) and transported from the home laboratory to the pre-flight preparation laboratories at the NOVESPACE premises in Bordeaux, France. After arrival, cells were stored at 36.5 °C overnight and used for the flight experiment on the following morning. A temperature of 36.5 °C was chosen instead of 37 °C to rule out any thermic activation of the cells caused by regulatory oscillation of the storage rack. For the flight day, the Nutrimix bags were placed in a solid plastic housing to create a double containment that prevents spillage of fluids in the aircraft in case of leakage of the hardware system. Rapid lysis of Jurkat T cells in the respective gravity phase was achieved by fast injection of five volumes of RLT buffer (Qiagen, Germany) and mixing by inverting the samples three times immediately. The 1 g in-flight controls were performed 5 min before the first parabola, and the 1.8 g samples were lysed directly before the microgravity phase of the first parabola. The microgravity samples were lysed directly at the end of the microgravity phase of the first parabola. After landing, 1 g ground controls were performed immediately using the same hardware inside the aircraft. Figure 2 shows a schematic overview of the individual lysis time points for the samples of different gravity phases. Post-flight, all samples were directly transported to the on-site laboratory where total RNA was purified. In total, 24 samples were obtained: 6 × 1 g ground controls, 6 × 1 g in-flight controls, 6 × 1.8 g and 6x µg.

### RNA isolation after the parabolic flight

After landing of the aircraft and transport of the samples to the laboratory facilities, the protective plastic housings were disassembled, the Nutrimix bags were gently agitated and the lysed cell solution was filled into a T75 straight neck cell culture flask. RNA was prepared with the RNeasy Maxi/Midi Kit following the manufacturer’s protocol (Qiagen, Germany): the cell solution was mixed for 10 s by vortexing and sheared by passing four times through a Ø 0.8 × 120 mm needle (B. Braun Melsungen, Germany) fitted to a sterile 50 ml syringe. About 50 ml of absolute ethanol was added, and precipitates were resuspended by vigorous shaking. A Qiavac 24 plus vacuum system (Qiagen, Germany) was prepared by placing 24 valves and sterile connective pieces on the Qiavac 24 plus vacuum manifold and an RNA maxi column (Qiagen, Germany) was attached to each connective piece. The system was set to a vacuum level of −200 mbar, and the RNA maxi columns were loaded with the lysed cell suspensions. Subsequently, the valves were closed, and the RNA maxi columns were centrifuged at 3220 g for 3 min at room temperature, and 15 ml of buffer RW1 (Qiagen, Germany) were carefully applied to the column to wash the membrane-bound RNA. After centrifugation at 3220 g for 7 min at room temperature, the flow through was discarded, and additional two washing steps were performed with 10 ml RPE buffer (Qiagen, Germany) followed by centrifugation at 3220 g for 3 min and 10 min at room temperature, respectively. The column-bound RNA was eluted by application of 600 µl of pre-warmed RNase-free water (Qiagen, Germany), incubation for 1 min at room temperature and centrifugation for 4 min at 3220 g again at room temperature. The elution step was repeated with the first eluate, the column was centrifuged for 7 min at 3220 g, and the purified RNA was stored in a sterile 1 ml cryotube either on dry ice or at approximately −150 °C in a Cryo Express dry shipper (CX-100, Taylor-Wharton, USA) prepared with liquid nitrogen. The RNA was stored at −80 °C until preparation for microarray analysis.

### TEXUS-51 suborbital ballistic rocket experiment

The TEXUS-51 suborbital ballistic rocket consisted of a two-stage VSB-30 rocket (S-30 solid rocket-stage engine with S-31 second-stage engine) and the payload (weight 390.4 kg, length 5083 mm). TEXUS-51 was launched on 23 April 2015 at 0935 hours from the ESRANGE (European Space and Sounding Rocket Range) Space Center near Kiruna, Sweden, north of the Arctic Circle. During the ballistic suborbital flight, an altitude of 258 km and 369 s of microgravity with a quality of better than 10^−4^ g was achieved. Further parameters include: first stage peak thrust acceleration of 8.1 g at 2.4 s, mean thrust acceleration of 5.1 g, first stage burnout at 12.1 s, engine separation at 13.4 s, second-stage peak thrust acceleration of 12.6 g at 34.9 s, mean thrust acceleration of 6.7 g, burnout at 43.2 s, spin at burnout of 2.8 Hz, yo-yo despin at 56.0 s, engine separation at 59.0 s, maximum residual reentry deceleration of 13.9 g at 486.6 s, heat shield release at 7.4 g and 596.5 s, main parachute release at 2.4 g and 623.4 s, sink rate of 7.6 m/s, impact after 884 s at 68°34.6395’ N 21°09.1263’ E. At ESRANGE, fully equipped laboratories enabled complete on-site preparation of the biological experiments, integration of the experiment into the payload platform 1 h before launch, and autonomous experiment execution in a programmed sequence. At the end of the free-fall period, the payload reentered the atmosphere and returned to the ground after parachute deployment at 5 km altitude and with a sink velocity of 8 m/s. One helicopter immediately recovered the experimental unit. The payload structure was recovered by a second helicopter and returned to the launch site within 1.5 h after liftoff. The general experimental composition consists of multiple sets of three syringes, filled with cell suspension (human Jurkat T cells), cell culture medium, and lysis solution (Trizol LS). The syringe system is constructed to allow the addition of an active ingredient (e.g., for activation) to the cells prior to lysis. As this mission was planned to investigate the effect of gravity alterations in basal cell homeostasis, only cell culture medium was filled into the syringe instead of an active ingredient. All three syringes were connected by a T-piece, while small plugs at the outlet ports prevented premature contact of the fluids. The syringe systems were housed in a temperature-controlled, vacuum-resistant container. The temperature-controlled syringe systems were placed at microgravity positions inside the payload structure, as well as on a centrifuge, which generates 1 g gravitational force as reference. Before launch and during flight, syringes were activated by a pneumatic system at pre-set time points. Several pre-flight tests and development tests were conducted: biocompatibility tests, chemical stability tests, culture medium optimization regarding buffer systems and supplements, sterilization tests, viability tests, and cell lysis tests (different lysis compounds and concentrations). The entire mission procedure was standardized and tested several times. Margins and possible holding times were determined. The experimental setup consisted of the baseline group (lysis at the onset of microgravity), the in-flight microgravity group (lysis after 5 min of microgravity and before reentry into the Earth’s atmosphere), the 1 g in-flight reference group (lysis after 5 min of 1 g centrifugation and before reentry into the Earth’s atmosphere), the 1 g ground control reference inside the experimental hardware, and the cell culture controls (lysis on ground). Each experimental group consisted of at least seven samples. Cells, medium, and lysis fluid (Trizol LS) syringes were prepared directly before the launch. All procedures started 7 h before launch. The experimental containers were integrated into the payload structure by a “late access” port between 01.15 and 00.45 hours before launch. Sample temperature was maintained at 36.5 ± 0.5 °C until lysis. On landing and payload recovery, the experimental containers were immediately removed and returned to the ESRANGE laboratory for further processing. The cell suspension was transferred from the syringes into sterile plastic reaction tubes, and cells were homogenized with subsequent isolation of RNA. The purified RNA was stored and transported on dry ice or in liquid nitrogen, and analyzed afterwards by means of genome-wide Affymetrix Expression Arrays.

### Experiment preparation and integration for TEXUS-51

Human Jurkat T cells were cultured in the ESRANGE laboratories on site. Cells were cultivated with a density of 0.2 × 10^6^ cells/ml, and the medium was exchanged every 48 h (see above). During the countdown phase, cells were visually inspected, harvested, the vital cell number was counted, and cells were pooled to a concentration of 5 × 10^7^ cells/ml. About 0.5 ml of cells (i.e., 25 million cells) was filled in sterile 3 ml plastic syringes shortly before the handover to the launch team. Additionally, a second set of syringes was filled with 0.3 ml of cell culture medium and a third set with 1 ml Trizol LS (Life Technologies, Germany) per sample unit. The three syringes with small plugs at the outlet ports were mounted on a sterilized plastic T-block with a connecting tubing system. This experimental unit was finally integrated into the automatically operated experiment system. The experiment units were prepared and were kept at 36.5 ± 0.5 °C until the integration into the payload of the rocket in the late access phase. During the experimental run, first 0.3 ml of medium and second 1 ml of Trizol LS were injected to the cell suspension at defined time points to lyse the cells and preserve the current status of differential gene expression. The sequential injection of fluids was performed for the different sample groups as shown in Fig. 2. Directly before the µg phase, a set of samples was lysed at the time point of 75 s after launch (baseline, BL), representing the effect of the hypergravity, the spin, and vibrations during the launch and rocket engine burn. Two further sets of samples were lysed at 375 s after launch, shortly before the end of the µg phase. During TEXUS-51, one sample group was installed on an integrated 1 g centrifuge, while the other group represented the microgravity samples. Additionally, 1 g ground controls, as well as cell culture controls were kept on ground in the incubator analogously to the µg sample group. In total, 39 samples were obtained after the TEXUS-51 rocket flight: 7 × 1 g ground cell culture controls, 7 × 1 g hardware controls (H/W), 9 × 1 g in-flight controls, 7x BL, and 9x µg. The sample groups are summarized in Table 1.

### RNA isolation after TEXUS-51 landing

Directly after landing, localization and recovery of the payload by helicopter, the experiment modules were dismantled and handed over for processing. The sample containing syringes were connected to a sterile 20G needle (B. Braun Melsungen, Germany), the 1.8 ml of cell suspension was sheared three times and distributed equally in two 2.0 ml reaction tubes. About 0.1 ml of chloroform (Sigma-Aldrich, Germany) was added, the homogenate was vortexed for 15 s and incubated for 5 min at room temperature before a 15 min centrifugation step at 11,000 g and 4 °C. The upper phase within both 2.0 ml tubes was transferred into a 15 ml tube, and 4 ml of RLT buffer (Qiagen, Germany), as well as 3 ml of absolute ethanol was added, and the suspension was mixed. About 4 ml of this solution was pipetted on an RNA Midi column (Qiagen, Germany), and centrifuged for 30 s at 3000 g and room temperature. The flow through was discarded and the residual 4 ml of RNA solution were loaded on the column and samples were centrifuged for 5 min at 3000 g at room temperature. Then, the columns were washed twice with 2.5 ml of RPE buffer (Qiagen, Germany), and centrifuged firstly for 2 min, and additionally for 5 min at 3000 g at room temperature. The RNA was eluted by the addition of pre-warmed 250 µl RNase-free water (Qiagen, Germany) to the column, incubation for 1 min at room temperature, and centrifugation for 3 min at 3000 g and room temperature. The flow through was loaded again onto the column, incubated for 1 min at room temperature, and centrifuged for 5 min at 3000 g and room temperature. The isolated RNA was transferred into sterile 1 ml cryotubes and stored and transported at −80 °C. After arrival in the home laboratory, samples were stored at −80 °C until the processing of the RNA for the microarray analysis.

### 2D clinostat and centrifuge ground experiments

Ground-based simulators of microgravity are valuable tools for preparing spaceflight experiments, but they also facilitate stand-alone studies and thus provide additional platforms for gravitational research.^62^ 2D clinorotation (DLR pipette clinostat, Institute for Aerospace Medicine, Gravitational Biology Group, Cologne, Germany) and centrifugation (pipette centrifuge, KEK GmbH, Bad Schmiedeberg, Germany) were used for simulated microgravity and hypergravity experiments, respectively. Human Jurkat T cells were prepared for both experimental setups from the same cell pool. Cells were centrifuged at 300 g for 5 min at room temperature and pooled to obtain a final concentration of 31.25 × 10^6^ cells/ml. About 0.8 ml of the cell suspension was filled in 1 ml pipettes (Becton Dickinson/Falcon), which corresponds exactly to 25 million cells in total and therefore to the experiment design of TEXUS-51. Two sterile rubber plugs were used to seal the pipettes and the samples were placed for 300 s either in the 2D pipette clinostat rotating with 60 rpm or the centrifuge set to 9 g at 37 °C (Fig. 1). The chosen 9 g hypergravity was in the range of the acceleration of the TEXUS-51 suborbital ballistic rocket (between 5.1 g for first stage mean thrust acceleration and 12.6 g for second-stage peak thrust acceleration). Additionally, pipettes filled with Jurkat cells were placed for the same time period at the base plate of the clinostat and used as 1 g hardware control exposed to all hardware effects, including potential vibration, but without clinorotation. A fourth set of samples representing a baseline directly before the experiment was prepared. Cells were aspirated in a pipette and directly emptied to reflect the effects of the filling procedure. Immediately after the respective incubation time, the cells were released in 1 ml Trizol LS and the suspension was homogenized with a sterile needle (0.8 × 80 mm; B Braun) and syringe. Total RNA was then purified according to the protocol applied also for the sounding rocket RNA samples. Six samples were analyzed per gravity condition with an Affymetrix microarray.

### Sample processing and microarray analysis for 23rd DLR PFC, TEXUS-51, clinostat, and centrifuge experiments

Gene expression profiling was performed using Affymetrix GeneChip Human Transcriptome Array 2.0 (Affymetrix United Kingdom Ltd., High Wycombe, United Kingdom) containing 44,699 protein coding genes and 22,829 non-protein coding genes. RNA quantity and quality were determined by measurement of concentration with absorbance at 260 and 280 nm (NanoDrop 2000c; Thermo, Fisher Scientific, Bonn, Germany) and by means of an Agilent 2100 Bioanalyzer with an RNA 6000 Nano kit and 2100 Expert software (version B.02.07) (all Agilent Technologies Deutschland GmbH, Boeblingen, Germany) at the Core Facility Genomics of the Medical Faculty Muenster (Muenster, Germany). Only high-quality RNA with 260/280 nm ratios between 1.97 and 2.04 and RNA integrity numbers >8.2 was used for further microarray analysis. The fragmented and biotinylated DNA targets were prepared according to the standard Affymetrix WT PLUS Reagent Kit protocol (Affymetrix GeneChip WT PLUS Reagent Kit, 902280) from 100 ng total RNA starting material and 5.5 µg complimentary DNA intermediate product. DNA targets were hybridized for 17 h at 45 °C on GeneChip Human Transcriptome Arrays 2.0. GeneChips were washed and stained in the Affymetrix Fluidics Station 450 according to the standard GeneChip Expression Wash, Stain and Scan protocol (Affymetrix GeneChip Wash, Stain and Scan Kit, 900720). Subsequently, the GeneChips were scanned using the Affymetrix 3000 7G scanner. For microarray data analysis, the Affymetrix Expression Console and Transcriptome Analysis Console was used. The robust multi-array averaging method was applied for background correction, quantile normalization, and probe summarization. After background correction, the base2 logarithm of each background-corrected perfect-match intensity was obtained. These background-corrected and log-transformed perfect-match intensities were normalized using the quantile normalization method. In the quantile normalization method, the highest background-corrected and log-transformed perfect-match intensity on each GeneChip is determined. These values were averaged, and the individual values were replaced by the average. This process was repeated with what were originally the second highest background-corrected and log-transformed perfect-match intensities on each GeneChip, the third highest, etc. Gene expression differences were determined by applying an analysis of variance.

### Statistical analysis

Log2 values of the measured fluorescent intensities of the investigated genes were retrieved from Affymetrix expression and transcriptome analysis console. Replicate values of the different experimental groups were compared by one-way analysis of variance and Tukey’s multiple comparison test with 95% confidence intervals using GraphPad Prism 5.04 (GraphPad Software Inc., San Diego, California, USA). Values were considered to be significantly different, if the *p*-value was <0.05. Values are expressed as means with SEM.

### DAVID

The gene annotation enrichment analysis was carried out by using DAVID^63, 64^ 6.8 and the gene ontology enrichment analysis and visualization tool GOrilla.^65, 66^

### Coefficient of variation value

Further, the coefficient of variation expressed in percent was calculated, also known as relative standard deviation. It equals the standard deviation divided by the mean.

### RefFinder

Finally, RefFinder,^25^ which is a comprehensive web-based tool that integrates geNorm,^26^ NormFinder,^27^ BestKeeper,^28^ and the comparative delta Ct method,^29^ was applied to determine genes with the most stable expression under various experimental conditions. RefFinder combines the four above algorithms and ranks the candidate genes by calculating the geometric mean of all four approaches. The lowest geometric mean value is the most stable gene.

### Data availability

The data sets generated during and/or analyzed during the current study are available in the GEO (Gene Expression Omnibus) repository (www.ncbi.nlm.nih.gov/projects/geo), accession no. GSE101102.

## Electronic supplementary material

Supplementary Note for GOrilla gene cluster analysis identifies and visualizes enriched GO terms in ranked gene lists
