# Supplementary material for: Stability of gene expression in human T cells in different gravity environments is clustered in chromosomal region 11p15.4
Source: NPJ Microgravity. 2017 Aug 31;3:22. doi: 10.1038/s41526-017-0028-6 (PMC5579209; doi:10.1038/s41526-017-0028-6)
Supplement: Supplementary file 1 — Supplementary Note for GOrilla gene cluster analysis identifies and visualizes enriched GO terms in ranked gene lists [file 41526_2017_28_MOESM1_ESM.pdf]

Stability of gene expression in human T cells in different gravity environments is clustered in chromosomal region 11p15.4

Supplementary Note

For gene cluster analyses, the tool GOrilla was used, which identifies and visualizes enriched GO terms in ranked gene lists. Fig. S1 and S2 show the GOrilla results derived from the comparison of the real microgravity platforms parabolic flight and sounding rocket as well as for the comparison of all three platforms. Analysis of biological process genes revealed an over-representation of GPCR signaling and sensory perception genes (fig. S1b, S2b). In the comparison of the three platforms the topic "cellular component" is additionally among the significant hits. The results fit well in the classification of "intrinsic and integral components of membrane" (fig. S1c), to the topics of "G protein coupled receptor activity and signaling" (fig. S1-2a,b). This analysis shows, that a large part of transcripts remains unchanged under altered gravity conditions and that the major part of the involved genes play a role in GPCR activity and signaling.

Figure S1. Gene ontology enrichment analysis (GOrilla) for ground-based facilities (GBFs), parabolic flight campaign (PFC), and sounding rocket experiment data. The intersection of transcripts with stable expression values obtained for the comparison of all three platforms was used in GOrilla to search for significant enriched GO features. Within the groups: biological process (**a**), molecular function (**b**) and cellular component (**c**) significant hits for the following categories were obtained: G-protein coupled receptor signaling pathways, sensory perception of smell, G-protein coupled receptor activity, olfactory receptor activity, and intrinsic and integral components of membranes.

Figure S2. Gene ontology enrichment analysis (GOrilla) for parabolic flight campaign (PFC), and sounding rocket experiment data. The intersection of transcripts with stable expression values obtained for the comparison of the real microgravity platforms PFC and sounding rocket was used for the GO enrichment analysis with GOrilla. Significant hits were obtained for the groups: biological process (**a**) and molecular function (**b**). These hits are coincident with the results obtained in the analysis for all three platforms.

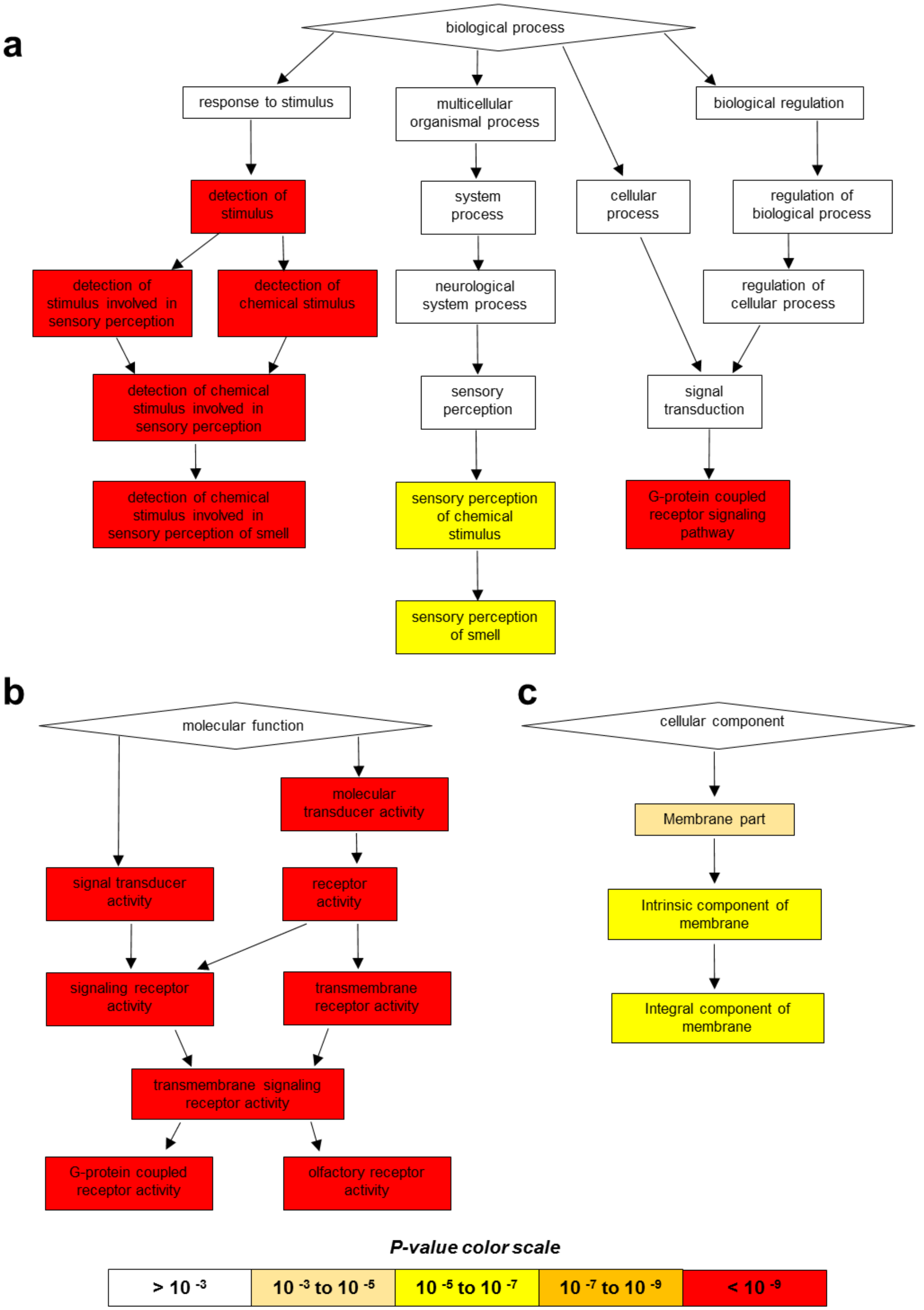
a
biological process
response to stimulus
multicellular organismal process
biological regulation
detection of stimulus
system process
cellular process
regulation of biological process
detection of stimulus involved in sensory perception
dectection of chemical stimulus
neurological system process
regulation of cellular process
detection of chemical stimulus involved in sensory perception
sensory perception
signal transduction
detection of chemical stimulus involved in sensory perception of smell
sensory perception of chemical stimulus
G-protein coupled receptor signaling pathway
sensory perception of smell
b
molecular function
molecular transducer activity
signal transducer activity
receptor activity
signaling receptor activity
transmembrane receptor activity
transmembrane signaling receptor activity
G-protein coupled receptor activity
olfactory receptor activity
c
cellular component
Membrane part
Intrinsic component of membrane
Integral component of membrane
P-value color scale
> 10 -3
10 -3 to 10 -5
10 -5 to 10 -7
10 -7 to 10 -9
< 10 -9

Fig. S1

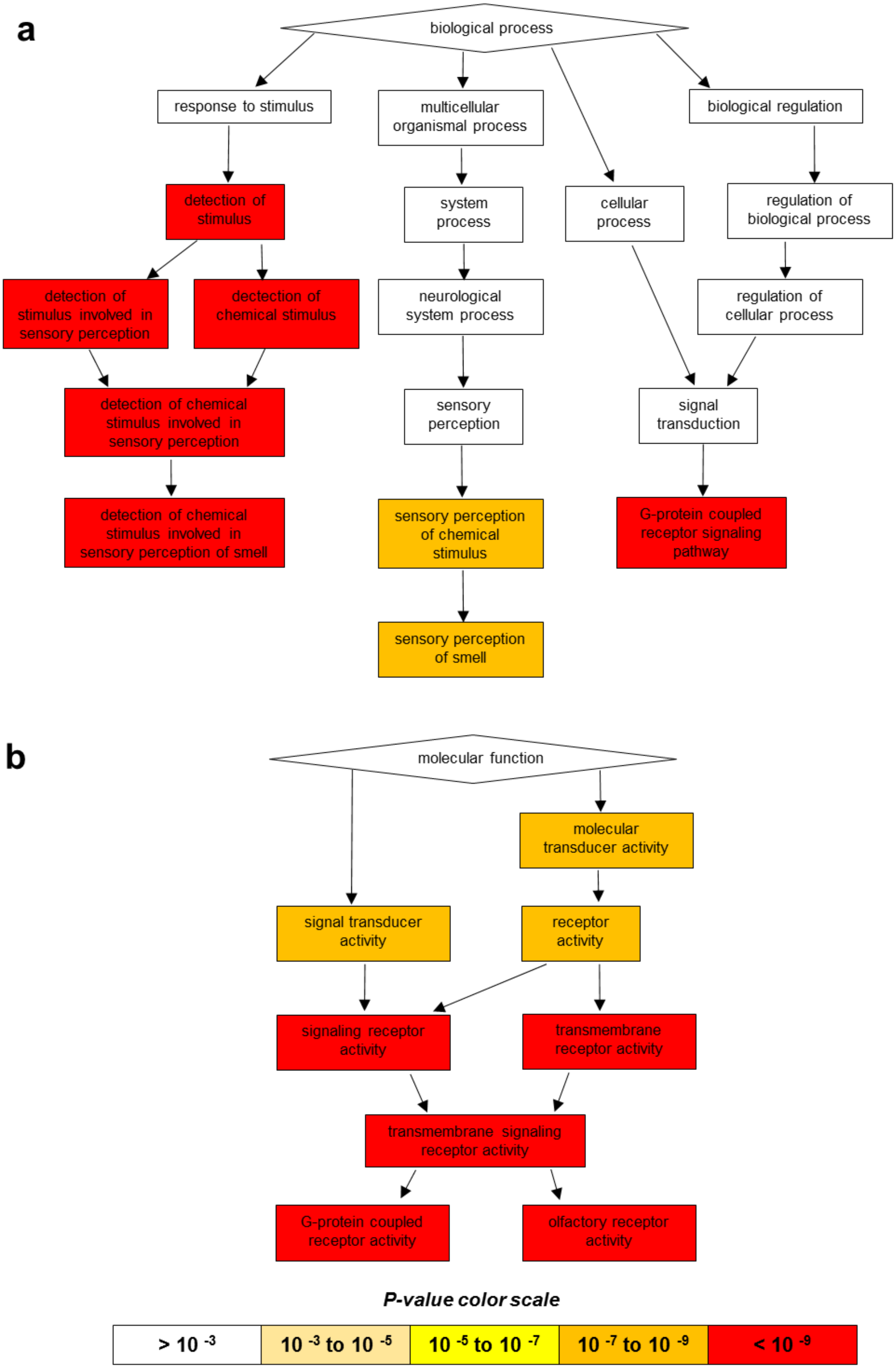
a
biological process
response to stimulus
multicellular organismal process
biological regulation
detection of stimulus
system process
cellular process
regulation of biological process
detection of stimulus involved in sensory perception
dectection of chemical stimulus
neurological system process
regulation of cellular process
detection of chemical stimulus involved in sensory perception
sensory perception
signal transduction
detection of chemical stimulus involved in sensory perception of smell
sensory perception of chemical stimulus
G-protein coupled receptor signaling pathway
sensory perception of smell
b
molecular function
molecular transducer activity
signal transducer activity
receptor activity
signaling receptor activity
transmembrane receptor activity
transmembrane signaling receptor activity
G-protein coupled receptor activity
olfactory receptor activity
P-value color scale
> 10 -3
10 -3 to 10 -5
10 -5 to 10 -7
10 -7 to 10 -9
< 10 -9

Fig. S2
